# Towards intelligent air quality forecasting using integrated machine learning framework with variational mode decomposition and catboost feature selection

**DOI:** 10.1038/s41598-025-33785-y

**Published:** 2026-01-02

**Authors:** Iman Ahmadianfar, Zaher Mundher Yaseen, Haydar Abdulameer Marhoon, Bijay Halder, Mou Leong Tan, Huseyin Cagan Kilinc, Sani I. Abba, Salim Heddam, Leonardo Goliatt, Vahdettin Demir, Ahmed M. Al-Areeq

**Affiliations:** 1 Department of Civil Engineering, Behbahan Khatam Alanbia University of Technology, Behbahan, Iran; 2 Interdisciplinary Research Centre for Membranes and Water Security, King Fahd University of Petroleum & Minerals, 31261 Dhahran, Saudi Arabia; 3 Civil and Environmental Engineering Department, King Fahd University of Petroleum & Minerals, 31261 Dhahran, Saudi Arabia; 4 College of Computer Sciences and Information Technology, University of Kerbala, Karbala, Iraq; 5 Information and Communication Technology Research Group, Scientific Research Center, Al-Ayen University, Thi-Qar, Iraq; 6 Department of Earth Sciences and Environment, Faculty of Science and Technology, Universiti Kebangsaan Malaysia, 43600 UKM, Bangi, Selangor Malaysia; 7 GeoInformatic Unit, Geography Section, School of Humanities, Universiti Sains Malaysia, Minden, 11800 Penang Malaysia; 8 Civil Engineering Department, Istanbul Aydın University, Istanbul, Turkey; 9 Department of Civil Engineering, Prince Mohammad Bin Fahd University, 31952 Al Khobar, Saudi Arabia; 10 Faculty of Science, Agronomy Department, Hydraulics Division University, 20 Août 1955, Route El Hadaik, BP 26, Skikda, Algeria; 11 Department of Computational and Applied Mechanics, Federal University of Juiz de Fora, Juiz de Fora, Brazil; 12 Dept. of Civil Engineering, KTO Karatay University, 42020 Konya, Turkey

**Keywords:** Air quality, Locally weighted, Kernel extreme learning machine, Multivariate variational mode decomposition, Catboost method, Applied mathematics, Computational science, Atmospheric chemistry, Environmental monitoring

## Abstract

Predicting air pollution is crucial in improving air quality (AQ), which consequently provides benefits to the ecosystems and human health. AQ predictions often make use of Machine Learning (ML) approaches; nevertheless, these methods are not without their limitations. The main contribution of this research is to develop an efficient framework using machine learning (ML) for forecasting daily air quality metrics for Sulfur dioxide (SO_2_) and Nitrogen dioxide (NO_2_) in Changping, China. The suggested ML method is based on a set of local weights and a kernel extreme learning machine (LWKELM) model integrated with an efficient feature selection, the Catboost method, to extract influential input variables. Additionally, the input variables, collected from 2013 to 2017, are decomposed using multivariate variational mode decomposition (MVMD), which enhanced the predicting accuracy. Furthermore, the interior search algorithm (ISA), a robust optimization strategy, is a possible way of optimizing the models’ hyperparameters. The results of the developed model was compared with the four other reliable ML approaches, including the locally weighted linear regression (LWLR), gaussian process regression (GPR), KELM, and multivariate adaptive regression spline (MARS) models. Based on the results, the proposed model demonstrates superior performance across statistical metrics for both parameters (NO₂: *R* = 0.978, RMSE = 0.537 and SO₂: *R* = 0.974, RMSE = 1.965) compared to alternative models. The MVMD-LWKELM-ISA model delivers highly accurate one-day-ahead forecasts for SO_2_ and NO_2_ and stands out as the most effective and intelligent approach for forecasting these daily parameters.

## Introduction

Recently, researchers have paid much attention to the harmful and adverse effects of air pollution on human health and work. The issue of air pollution has attracted numerous researches conducted by researchers on the health effects of air pollution and other aspects^1–3^. Laboratory research has demonstrated that environmental pollutants may cause bronchitis, asthma, and emphysema among other breathing disorders^4^. Accordingly, the authority carrying out environmental oversight of different countries is focusing on constructing automatic monitoring stations to monitor several air pollution indicators (API) such as particulate matter (PM_10_ and PM_2.5_), nitrogen dioxide (NO_2_), sulfur dioxide (SO_2_), ozone (O_3_), etc. ^4^. Such pollutants have raised a lot of concern in the past few years because of the negative impacts of their existence on the health of the society^2^. This is the reason why the air quality forecasting research has to be considered within the context of the current environmental condition to see how air pollution in the ambient air can be minimized.

The problems of time series forecasting of air pollutants have been addressed in several studies^2,5–8^. A neuro-fuzzy inference system (ANFIS) was proposed by Prasad et al. (2016) to forecast five daily APIs inside a Megacity’s atmospheric atmosphere, namely PM_10_, NO_2_, SO_2_, O3, and CO ^9^. According to the findings, the proposed model accurately forecasted multiple APIs. Researchers employed the support vector regression (SVR), MLP, vector autoregressive moving average (VARMA), and autoregressive integrated moving average (ARIMA) to forecast the PM_10_ from one to seven months ahead^10^. According to simulations, the SVR model performed better than any other model when forecasting one and seven months ahead of PM_10_. Another research focused on modeling the NO_2_ and SO_2_ in the Central China region^2^. They suggested a concept that they called the multi-step hybrid model, which could be summed up as the following three steps: (i) decomposition of NO_2_ and SO_2_ time-series into low-frequency (LF) and high-frequency (HF), (ii) modeling the LF and HF sequences using SVR coupled with grey wolf optimizer (GWO) and cuckoo search (CS), correspondingly; (iii) sum up the LF and HF data forecasting results as a final prediction result for NO_2_ and SO_2_. Based on comparisons with other methods, the proposed hybrid model with two steps was more accurate.

Harishkumar et al. (2020) investigated six ML models (gradient boosting regressor (GBR), linear regression (LR), random forest (RF), K neighbors regressor, multi-layer perceptron (MLP), and decision tree regressor CART) for predicting PM_2.5_ parameter^11^. The authors demonstrated that the GBR model performed much better in predicting PM_2.5_ levels. Shams et al. (2021) applied the MLP and multiple linear regression (MLR) to predict SO_2_ in Tehran^12^. The MLP model that was proposed in this study could be used to aid in, analyze, and enhance the process of forecasting air pollution and the management of air quality. The findings of this research highlighted the significance of using the modeling and the use of artificial neural networks (ANN) when providing management plans to cut down on urban pollution. Research reported introduced a forecasting model based on the long short-term memory (LSTM) deep learning optimized by genetic algorithm (GA) to forecast four air pollutants parameters (i.e., PM_10_, PM_2.5_, CO, and NOX)^13^. They indicated that the proposed model was more precise and faster than the ML methods and the LSTM model, at the same time. Almalawi et al. (2022) used the SVR, linear regression (LR), and gradient-boosted decision tree (GBDT) models to forecast the air qualify index (AQI) over the next 5 h^3^. It is found that the GBDT method can achieve the least error compared with other methods.

According to the literature review, the main drawbacks of previous works can be described as follows,

i. The majority of the previous research stuck to the tried-and-true approaches of classical ML. For instance, the SVR, MLP, GBR, and ANN were used in their standard forms. Consequently, these techniques demonstrated limited ability in detecting complex patterns that existed within the data, thus affecting their prediction accuracy and analytical effectiveness.
ii. The previous studies commonly have not been applied feature selection methods to identify the important feature. The techniques of feature selections reduce the dimensions of the problem and remove features that are irrelevant.
iii. Multivariate decomposition techniques were rarely employed in previous research. These techniques break down features into low and high-frequency components that may potentially improve the performance and predictive accuracy of ML algorithms.

Based on the above shortcoming, this work develops an efficient locally weighted kernel extreme learning machine (LWKELM) optimized utilizing an efficient optimization method, interior search algorithm (ISA)^14^, designed to forecast NO_2_ and SO_2_ air quality parameters. To enhance the accuracy of predictions, the proposed LWKELM model employs a set of weighting factors on the input variables. The least squares (LS) approach is used to get these weights. The ISA technique is used to derive optimal control parameters of the LWKELM model. The ISA method employs an efficient global and local search strategy to rapidly and accurately converge toward the global optima. In this research, to predict NO_2_ and SO_2_, the input variables are ozone (O_3_), carbon monoxide (CO), air temperature (T), PM_10_, PM_2.5_, dew point temperature (DPT), Pressure (P), and wind speed (WS) at a daily time step from Beijing Changping station, China. In addition, to increase the forecasting accuracy, the multivariate variable mode decomposition (MVMD)^15^ is used as a decomposition method. It is known that the Catboost model serves as a reliable model to filter influential input variables for all models because of its capabilities of capturing the strong non-linear correlation that exists between inputs and outputs. The main aim of this research is to promote an efficient ML method regarding LWKELM-ISA for the forecasting of NO_2_ and SO_2_ daily. We use four alternative hybrid models (i.e., KELM-ISA, GPR-ISA, MARS-ISA, and LWLR-ISA) as a means to confirm the stability of the proposed model and to demonstrate that LWKELM-ISA has the superior performance as a framework for utilizing air quality forecasting to the fullest extent possible.

Every section in this paper has its structure, the first section is the introduction. The section “Materials and methods” outlines the material and methods that were used in this research. In the section “Development of MVMD-based models”, the development of MVMD-based models is described. The section “Results and discussions” explains how MVMD-based models are constructed and the results of any ML models. The final section is a conclusion of the key findings of this paper.

## Materials and methods

### Extreme learning model

The development of the ELM concept was credited to Huang et al.^16^ as pioneers. The method just needs one step to calculate the output weights; hence there is no need for any iteration at any stage of the procedure. The main advantages of this method are that it is easy to implement and simple to learn. Researchers have recently reported the modification of the ELM, kernel ELM (KELM) model, which has been reported for the first time in the literature^17^. KELM makes use of a function that brings together the best features of ELM and kernel analysis. A hidden node ELM with *N* hidden nodes could be derived from the following equation, which shows the output from a hidden node ELM:

A hidden-node ELM with *N* hidden nodes can be expressed by the following equation, which represents the output of the hidden layer:

1 $$\:{u}_{i}={\sum\:}_{n=1}^{N}{\theta\:}_{n}g({w}_{n}{x}_{i}+{c}_{n}),\:\:\:\:\:\:i=\mathrm{1,2},\:\dots\:,\:L$$

where $\:{u}_{i}$ expresses the estimated output, $\:{w}_{n}$ is the weight vector connecting the input layer to the $\:n$-th hidden node, $\:{x}_{i}$ indicates the input parameters, $\:{\theta\:}_{n}$ denotes the weight vector (W) between the hidden layer (HL) and the output layer, $\:{c}_{n}$ expresses the HL bias, and $\:g$ indicates the activation function for the HL. Equation (1) can be reformulated as,

2 $$\:u=\theta\:.G$$

where $\:G$ denotes the matrix of HL output, and $\:\theta\:$ explains the weight vector (W) between the hidden layer (HL) and the output layer, expressed as,

3 $$\:G = \left[ {\begin{array}{*{20}c} {g\left( {w_{1} x_{1} + c_{1} } \right)} & {\: \ldots \:} & {\:g\left( {w_{N} x_{1} + c_{N} } \right)} \\ {\: \vdots } & {\: \ldots \:} & {\: \vdots } \\ {\:g\left( {w_{1} x_{L} + c_{1} } \right)} & {\: \ldots \:} & {\:g\left( {w_{N} x_{L} + c_{N} } \right)} \\ \end{array} } \right]$$

Equations (1)–(3) describe how ELM maps inputs to outputs through a hidden layer. In Eq. (1), the predicted output $\:{u}_{i}$is computed as a weighted sum of the activations from $\:N$hidden nodes, where each node applies an activation function $\:g(\cdot\:)$ to a linear combination of the input $\:{x}_{i}$and its bias $\:{c}_{n}$. Equation (2) rewrites this in matrix form, $\:u=\theta\:G$, where $\:G$ (Eq. 3) is the hidden-layer output matrix collecting all hidden-node activations for all training samples. This formulation highlights that ELM training reduces to solving for the output weights $\:\theta\:$ in a linear system, while KELM replaces $\:G$ with a kernel-based mapping to efficiently capture nonlinear patterns.

A fitness function ($\:Ft$) needs to be minimized by the ELM to determine the best value for the weight vector, as can be seen below:

4 $$\:Ft={\sum\:}_{i=1}^{L}{\left({\sum\:}_{n=1}^{N}{\theta\:}_{n}g({w}_{n}{x}_{i}+{c}_{n})-{Y}_{i}\right)}^{2}\:\:\:\:\:\:\:\:\:\:$$

where$\:{Y}_{i}$ indicates the output vector.

Applying the extended inverse principle, Eq. (3) can be reformulated as,

5 $$\:\theta \: = G^{\dag } Y$$

where G† expresses the Moore-Penrose inverse matrix of $\:G$. Huang developed the KELM kernel function, which is based on the orthogonal projection technique and ridge regression theory. This function aims to improve the reliability of ELM by adding a constant $\:I/D$ to the calculation of $\:\theta\:$. Adding the KELM kernel function allowed Huang to improve the ELM method. The parameter $\:\theta\:$ can be calculated by Eq. (6).

6 $$\:\theta\:={\left({G}^{T}G+\frac{I}{D}\right)}^{-1}{G}^{T}Y$$

where *I* indicates the identity matrix. Consequently, it is determined that the ELM’s output function is,

7 $$\:u=g\left(x\right)\theta\:=g\left(x\right){\left({G}^{T}G+\frac{I}{D}\right)}^{-1}{G}^{T}Y$$

### Kernel extreme learning machine

Though the ELM performs well, its randomized behavior is a major downside. Thus, Huang thought of the KELM as a means of resolving this issue. The KELM replaces the activation function *G* with the kernel matrix (KM). It is possible to write an equation that is related to the kernel matrix in the following manner:

8 $$\:\varphi\:={G}^{T}G:\:{\varphi\:}_{i,l}=g\left({x}_{i}\right)g\left({x}_{i}\right)=Krn({x}_{i},{x}_{l})$$

Using Eq. (9), it is possible to calculate the output function of KELM.

9 $$u = \left[ {\begin{array}{*{20}c} {Krn\left( {x,x_{1} } \right)} \\ {\: \vdots } \\ {\:Krn\left( {x,x_{K} } \right)} \\ \end{array} } \right]\left( {\phi \: + \frac{I}{D}} \right)^{{ - 1}} Y$$

This study makes use of the wavelet kernel function (WavKr), a special kind of kernel function. A specific form of kernel function called the wavelet kernel function (WavKr) is used in this investigation. Here is how this function is written down in mathematical terms:

10 $$Krn\left( {x_{i} ,x_{l} } \right) = {\mathrm{cos}}\left( {\nu \:.\frac{{\left\| {x_{i} - x_{l} } \right\|^{2} }}{{\rho \:}}} \right).{\mathrm{exp}}\left( { - \frac{{\left\| {x_{i} - x_{l} } \right\|^{2} }}{{\mu \:}}} \right)$$

where $\:\nu\:$, $\:\rho\:$, and $\:\mu\:$ are the control parameters of the WavKr in the KELM approach.

Previous studies have shown that adjusting KELM’s parameters ($\:\nu\:$, $\:\rho\:$, and $\:\mu\:$) significantly affects the model’s performance^18,19^. This highlights the critical need of quickly determining appropriate values for these parameters. The major focus of this study has been on optimizing KELM parameters using the proposed ISA approach. Pollutants like SO_2_ and NO_2_ have been predicted using the resulting KELM-ISA model.

### The proposed LWKELM model

An enhanced KELM model is provided in this subsection using locally weighted linear regression (LWLR)^20^ called locally weighted KELM (LWKELM). Using the multivariate linear regression (MLR) method, the LWLR is provided in this study. A dependent parameter (*u*) could be calculated by establishing a linear equation with more than two independent parameters (*x*).

11 $$\:{u}_{i}={\beta\:}_{i0}+{\sum\:}_{n=1}^{N}{\beta\:}_{in}{x}_{in}+{\epsilon}_{i}$$

where ($\:{\beta\:}_{i0},{\beta\:}_{i1},\dots\:,{\beta\:}_{in}$) denotes the regression parameters calculated by applying the least square (LS) technique, $\:{\epsilon}_{i}$ indicates the error parameter, and *L* demonstrates the maximum number of the input dataset. Equation (12) offers a specification for a fitness function that will be used to identify the line that yields the best fit to the measured dataset ($\:{Y}_{n}$) utilizing the MLR technique.

12 $$\:Minimize\:\:\:Fit=\frac{1}{2L}{\sum\:}_{i=1}^{L}{\left({\beta\:}_{i0}+{\sum\:}_{n=1}^{N}{\beta\:}_{in}{x}_{in}+{\epsilon}_{i}-{Y}_{n}\right)}^{2}$$

Equation (12) is defined in a matrix framework as $\:{\left(X\beta\:-Y\right)}^{T}\left(X\beta\:-Y\right)$. By derivation of the matrix based on $\:\beta\:$, $\:{X}^{T}\left(X\beta\:-Y\right)$ is calculated. Let the matrix equal zero, and then solve for to get the LS:

13 $$\:\beta\:=({X}^{T}X{)}^{-1}{X}^{T}Y$$

where $\:X$ expresses the matrix of input parameters.

Using a weight function (WF), the LWLR can describe the relationship between the training dataset and the prediction.

14 $$\:Minimize\:\:\:\:\:Fit=\frac{1}{2L}{\sum\:}_{i=1}^{L}{\omega\:}_{i}{\left({\beta\:}_{i0}+{\sum\:}_{n=1}^{N}{\beta\:}_{in}{x}_{in}+{\epsilon}_{i}-{Y}_{n}\right)}^{2}\:\:$$

where $\:\omega\:$ denotes the WF matrix.

Equation (14) is defined in a matrix framework as $\:{\left(X\beta\:-Y\right)}^{T}\omega\:\left(X\beta\:-Y\right)$. In order to get the most accurate results that can be obtained from Eq. (14), it is essential that the derivative of Fit concerning $\:\beta\:$ must be equal to zero.

15 $$\:\frac{\partial\:Fit\left(\beta\:\right)}{\partial\:\beta\:}={X}^{T}\omega\:X\beta\:-{X}^{T}\omega\:Y=0$$

and

16 $$\:\beta\:=({X}^{T}\omega\:X{)}^{-1}\:{X}^{T}\omega\:Y$$

To promote the precision of the proposed LWKELM’s forecasts, the value of $\:\beta\:$ determined by Eq. (16) has been included into the kernel function. Therefore, Eq. (8) can be reformulated as,

17 $$\:\varphi\:=Krn(\beta\:{x}_{i},{\beta\:x}_{l})$$

Additionally, Eq. (9) is rewritten as, in order to boost the model’s performance even further:

18 $$u = \left[ {\begin{array}{*{20}c} {Krn\left( {x,x_{1} } \right)} \\ {\: \vdots } \\ {\:Krn\left( {x,x_{L} } \right)} \\ \end{array} } \right]\left( {\varphi \: + \frac{I}{{\eta \: \times \:D}}} \right)^{{ - 1}} Y$$

where $\:\eta\:$ indicates a penalty coefficient, which is achieved based on Zhang and Luo^21^ as follows:

19 $$\:\eta \: = \frac{{2L}}{{\left\| Y \right\|}}$$

where $\:L$ expresses the length of the dataset.

### ISA method

In this research, the interior search algorithm (ISA)^14^ is used to derive the optimal values of control parameters of ML models. The ISA is a population-based optimization algorithm, and has the ability to solve the optimization problems with great efficiency. The proposed algorithm was inspired by the behavior of an interior decorator and designer^14^. There are two primary operators for the ISA, (1) composition operator (CO), and (2) mirror operator (MO). For the purpose of seeking globally in the solution space, we use CO, while for locally searching in feasible space, MO is adopted. The ISA is broken down into the following stages.

*Stage 1*. The initial solutions in the ISA can be generated by Eq. (20).

20 $$\:{x}_{k\mathrm{,}j}^{g}=LW+\left(UP-LW\right)\times\:{r}_{1},\:\:\:\:\:\:k=1,\:2,\:\dots\:,\:K,\:\:\:j=1,\:2,\:\dots\:,\:D$$

where $\:LW$and $\:UP$ express the lower and upper bound, correspondingly, $\:{r}_{1}$is a random amount in the interval [0, 1], and $\:K$ and $\:D$ indicate the population size and the dimension, correspondingly.

*Stage 2*. The best solution ($\:{x}_{best\mathrm{,}j}^{g}$) is selected based on its objective function at each iteration.

*Stage 3*. All solutions can be divided into the composition and mirror parts utilizing the $\:\delta\:=\frac{g}{Maxg}$ parameter, where $\:g$ expresses the iteration number and $\:Maxg$ expresses the maximum number of iterations. If $\:rand\le\:\delta\:$, the new solution can be created employing the MO; otherwise, the new solution can be created by the CO.

*Step 4*. The solution created by CO can be randomly generated utilizing Eq. (21).

21 $$\:{x}_{k\mathrm{,}j}^{g}={x}_{min\mathrm{,}j}^{g}+({x}_{max\mathrm{,}j}^{g}-{x}_{min\mathrm{,}j}^{g})\times\:{r}_{2}$$

where $\:{x}_{min\mathrm{,}j}^{g}$and $\:{x}_{max\mathrm{,}j}^{g}$ express the minimum and maximum bounds of the CO in the *g*th iteration, and $\:{r}_{2}$denotes a random amount in the interval [0, 1].

*Step 5*. The solution generated by the MO is created based on the current solution and the best solution at each iteration. Therefore, the MO solution can be achieved by Eq. (22).

22 $$\:{x}_{MO\mathrm{,}j}^{g}={r}_{3}\times\:{x}_{k\mathrm{,}j}^{g}+(1-{r}_{3})\times\:{x}_{lbs\mathrm{,}j}^{g}$$

where $\:{r}_{3}$ denotes a random amount in the interval [0, 1], and $\:{x}_{lbs\mathrm{,}j}^{g}$ expresses the best solution obtained at each iteration. The new solution is achieved by utilizing the MO as

23 $$\:{x}_{new\mathrm{,}j}^{g}=2{x}_{MO\mathrm{,}j}^{g}-{x}_{k\mathrm{,}j}^{g}$$

*Step 6*. In the case of the local search, the location of the optimal solution shifts subtly by the equation that is shown below.

24 $$\:{x}_{lbs\mathrm{,}j}^{g}={x}_{lbs\mathrm{,}j}^{g}+randn\times\:\gamma\:$$

where $\:randn$ expresses a normally distributed random amount, and $\:\gamma\:$ denotes a positive parameter, defined as,

25 $$\:\gamma\:=0\mathrm{.}01\times\:(UP-LW)$$

### Filtering dataset

The precision of prediction algorithms can be reduced if a large number of inputs are used in the model development procedure. In general, there are a number of approaches that may be used to prioritize the inputs. Principal component analysis, correlation, and autocorrelation are the three most used statistical procedures. However, these techniques are often used for input-output interactions that are linear^5^. In this regard, these methods are widely employed for linear interactions between inputs and outputs.

Consequently, to increase the accuracy of the model, this study employed the Catboost data filtering approach to pick the influential input variables. CatBoost employs ordered boosting with symmetric trees, which improves gradient estimation and generalization, and is well-suited to discovering nonlinear, interaction-driven importance among predictors. This method is a proper nonlinear input variables method of selection. More specifically, this strategy is used to choose the most crucial confounding factors of the variable under study. Therefore, Catboost can be potential option to rank the relevance of features using ordered boosting and symmetric trees to improve nonlinear dependency capture and generalization.

In many practical scenarios, Catboost can be used as a highly effective gradient-boosting machine-learning algorithm^22^. This algorithm can provide cutting-edge results in a wide range of situations. Catboost uses an aggregation of symmetric decision trees with symmetry structures to accomplish its high precision and fast speed in testing and training. Catboost uses ordered boosting instead of the conventional gradient estimation approach used in conventional gradient boosting algorithms, which simultaneously reduces the bias in gradient estimation and increases the generalization ability of the algorithm. Therefore, this study uses the Catboost method to determine the most important input parameters for the SO_2_ and NO_2_ parameter forecasting.

### Multivariate variational mode decomposition

Rehman and Aftab^15^ created the MVMD, a development of the variational mode decomposition (VMD)^23^. A one-dimensional input variable is known as $\:{x}_{t}$ is broken down using VMD into *N* different sub-signals referred to as the intrinsic mode functions (IMFs)^23^. Therefore, by adding the *N* IMFs, the original signal *x* could be reconstructed in a least-squares context:

26 $$\:{x}_{t}=\sum\:_{n=1}^{N}{Imf}_{n,t}$$

This method for multivariate signals is advanced by the MVMD. For example, the MVMD will derive *N* multivariate IMFs ($\:{Imf}_{n,t}$) from a matrix that contains *n* different multiple signals, where $\:{Imf}_{n,t}=\{{Imf}_{1,t},\:\dots\:,\:{Imf}_{N,t}\}$. To handle the problem of optimizing variational modes in the Fourier domain, a strategy known as the alternating direction method of multipliers (ADMM) is used^15^. This method produces the best possible set of multivariate deconstructed signals, where all signals have common characteristics like narrow bandwidths and central frequencies^15^. The MVMD is an excellent method for multivariate decomposition because of the following characteristics: (1) divides the modulated frequencies into multiple components; (2) capability of mode matching possessed by multivariate signals; (3) Identification of signals effectively; (4) signal decomposition using a quasi-orthogonal structure^15,24^. Therefore, MVMD, unlike VMD, jointly decomposes all inputs into aligned IMFs with common central frequencies, enhancing predictive accuracy for multivariate AQ forecasting. Readers interested in obtaining further information on MVMD might go to ^15^.

### Statistical metrics

The present section introduces six criteria to assess the capability of ML methods to forecast the AQ parameters (NO_2_ and SO_2_). These criteria include the root mean square error (RMSE), maximum absolute error (MaxAE), uncertainty coefficient at a95% confidence level (U_95%_), correlation coefficient (R), the index of agreement (IA), and Nash-sutcliffe efficiency (NSE), which are mathematically formulated as,

27 $$\:R=\frac{\sum\:_{l=1}^{N}\left({AQ}_{Mo,l}-\:{\stackrel{-}{AQ}}_{Mo}\right)\times\:\left({AQ}_{Fr,l}\:-{AQ}_{Fr}\:\right)\:}{\sqrt{\sum\:_{l=1}^{N}({{AQ}_{Mo,l}-\:{\stackrel{-}{AQ}}_{Mo})}^{2}\times\:\:\sum\:_{\mathrm{i}=1}^{\mathrm{N}}({{AQ}_{Fr,l}\:-\:{\stackrel{-}{AQ}}_{Fr}\:)}^{2}\:\:\:\:\:}\:\:}$$

28 $$\:NSE=1-\frac{\sum\:_{l=1}^{N}{({AQ}_{Mo,l}-{AQ}_{Fr,l})}^{2}}{\sum\:_{l=1}^{N}{({AQ}_{Mo,l}-{\stackrel{-}{AQ}}_{Mo})}^{2}}\:$$

29 $$\:RMSE=\sqrt{\frac{1}{N}\:\sum\:_{l=1}^{N}({AQ}_{Mo,l}-\:{AQ}_{Fr,l}{)}^{2}}$$

30 $$\:{I}_{A}=1-\frac{\sum\:_{l=1}^{N}{\left({AQ}_{Fr,l}-{AQ}_{Mo,l}\right)}^{2}}{\sum\:_{l=1}^{N}{\left(\left|\left({AQ}_{Fr,l}-{\stackrel{-}{AQ}}_{Fr}\right)\right|+\left|\left({AQ}_{Mo,l}-{\stackrel{-}{AQ}}_{Mo}\right)\right|\right)}^{2}},\:0<{I}_{A}\le\:1$$

31 $$\:{U}_{95\%}=1.96\sqrt{{SD}^{2}+{RMSE}^{2}}$$

where the $\:{AQ}_{Fr,l}$ and $\:{AQ}_{Mo,l}$ are the forecasted and measured values of the AQI. The averages of forecasted and measured values are specified by $\:{\stackrel{-}{AQ}}_{Fr}$ and $\:{\stackrel{-}{AQ}}_{Mo}$, correcpondingly. *N* denotes the dataset size, while SD indicates the standard deviation.

### Data collecting and pre-processing

**Fig. 1:**
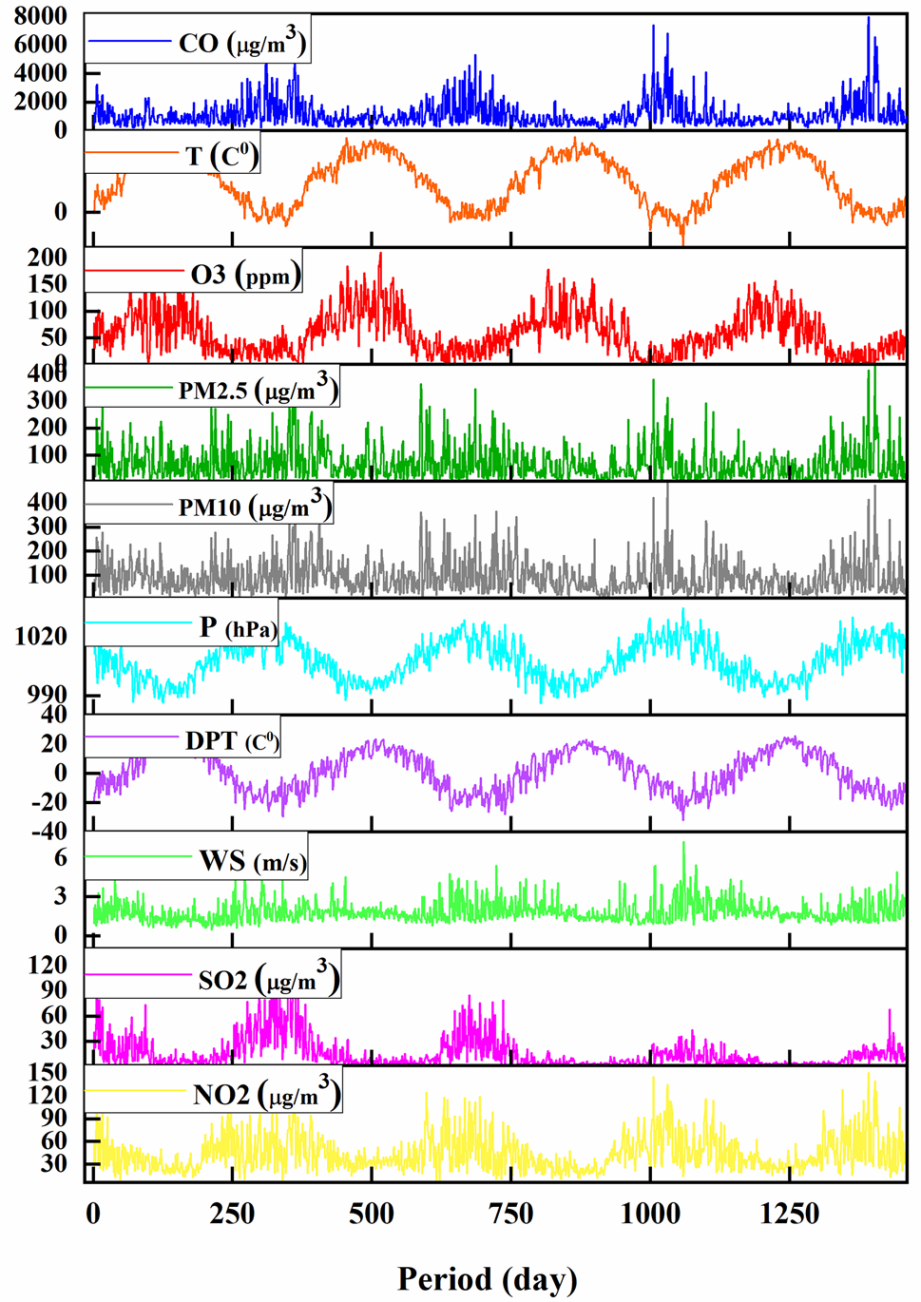
Time-series graph of all air quality parameters.

The data used in this study was given by the University of California, Irvine’s Center for Machine Learning and Intelligent Systems^25,26^. This research comprised data from 2013 to 2017 for the Chinese city of Changping (Latitude: 40°13’14.38"N, Longitude: 116°13’52.33"E). The main input parameters are PM_10_, PM_2.5_, CO, T, O3, WS, P, and DPT. The output variables are NO_2_ and SO_2_. Table 1 summarizes and analyzes the dataset, comprising the minimums, averages, maximums, skewnesses (SK), kurtosis (KU), and standard deviation (SD) amounts. Figure 1 shows a time series of the gathered data throughout time.

**Table 1:** Statistical characteristics of all API variables.

	SO_2_(µg/m^3^)	NO_2_ (µg/m^3^)	CO (µg/m^3^)	O_3_ (µg/m^3^)	T (^o^C)	P (mm)	DPT (^o^C)	WS (m/s)	PM_2.5_ (µg/m^3^)	PM_10_ (µg/m^3^)
Max	137.96	150.42	8112.5	210.33	32.37	1034.93	26	7.24	433.46	482.75
Avg	14.96	44.24	1160.04	58.04	13.67	1007.77	1.49	1.85	71.12	94.79
Min	1	6.83	125	2	-14.96	985.81	-32.23	0.45	4.25	5.63
SD	18.08	23.42	947.89	39.7	10.79	10.02	13.61	0.82	62.26	68.09
SK	2.52	1.25	2.66	0.75	-0.16	0.09	-0.12	1.69	1.79	1.61
KU	10.69	4.44	13.64	3.04	1.68	2.02	1.79	7.05	7.14	6.57

**Fig. 2:**
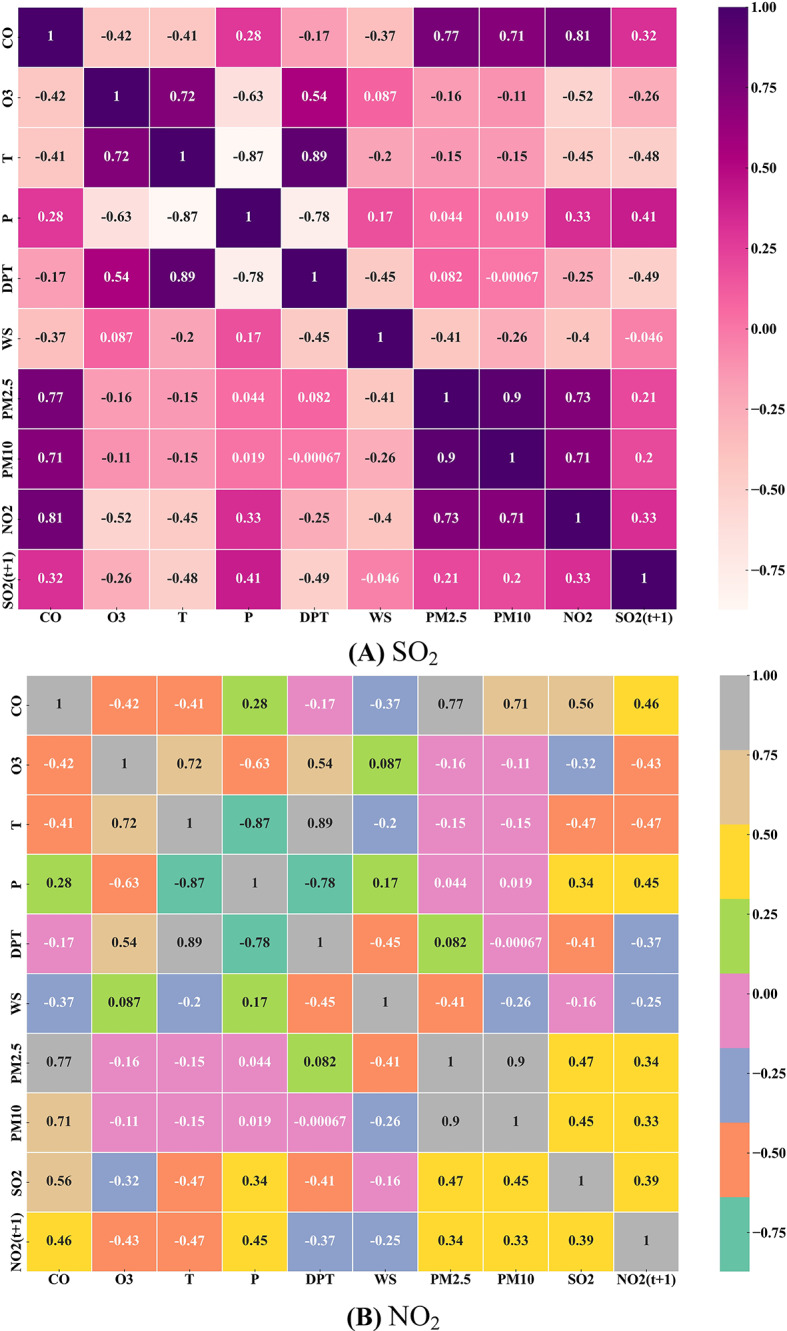
Correlation matrix between independent and dependent parameters for forecasting: **A** SO_2_ and **B** NO_2_ “Figure is generated using Python Software Foundation. (2016). Python (Version 3.x) [Computer software]. https://www.python.org/”.

Figure 2a and b illustrated the correlation matrix (CM) plot with all the input factors and the output factors (NO_2_ and SO_2_). The values in the graph show correlations between all parameters. According to this graph, SO_2_ (t + 1) has a strong correlation with NO_2_, P, T, CO, DPT, and O_3_. In addition, there is a high correlation between NO_2_ (t + 1) and CO, P, O_3_, T, SO_2_, PM_10_, and PM_2.5_.

## Development of MVMD-based models

Eight input factors, including PM_10_, PM2. 5, CO, O3, WS, P, T and DPT were applied to provide a 1-day ahead forecast of SO_2_ and NO_2_ over China. Here, all these models were developed using MATLAB 2020a to predict SO_2_ and NO_2_ one day in advance. The LWKELM-ISA, KELM-ISA, LWLR-ISA, MARS-ISA and GPR-ISA were the models proposed in this study. The stages of a model development are as follows.

### Phase 1: decomposition using MVMD

In this study, the time series quantities of the dataset (PM_10_, PM_2.5_, CO, O_3_, WS, P, T, and DPT) were utilized to forecast SO_2_ and NO_2_ variables at the next time step t + 1. It is important to remember that O3, PM_10_, PM_25_, CO, T, DPT, WS, P, NO_2_, and the SO_2_ time delays were used to anticipate the SO_2_ (t + 1) level. Also, to forecast the NO_2_, the time lags O3, PM_10_, PM25, CO, T, DPT, WS, P, SO_2_, and the NO_2_ time lags were applied to anticipate the NO_2_ (t + 1) level.

**Fig. 3:**
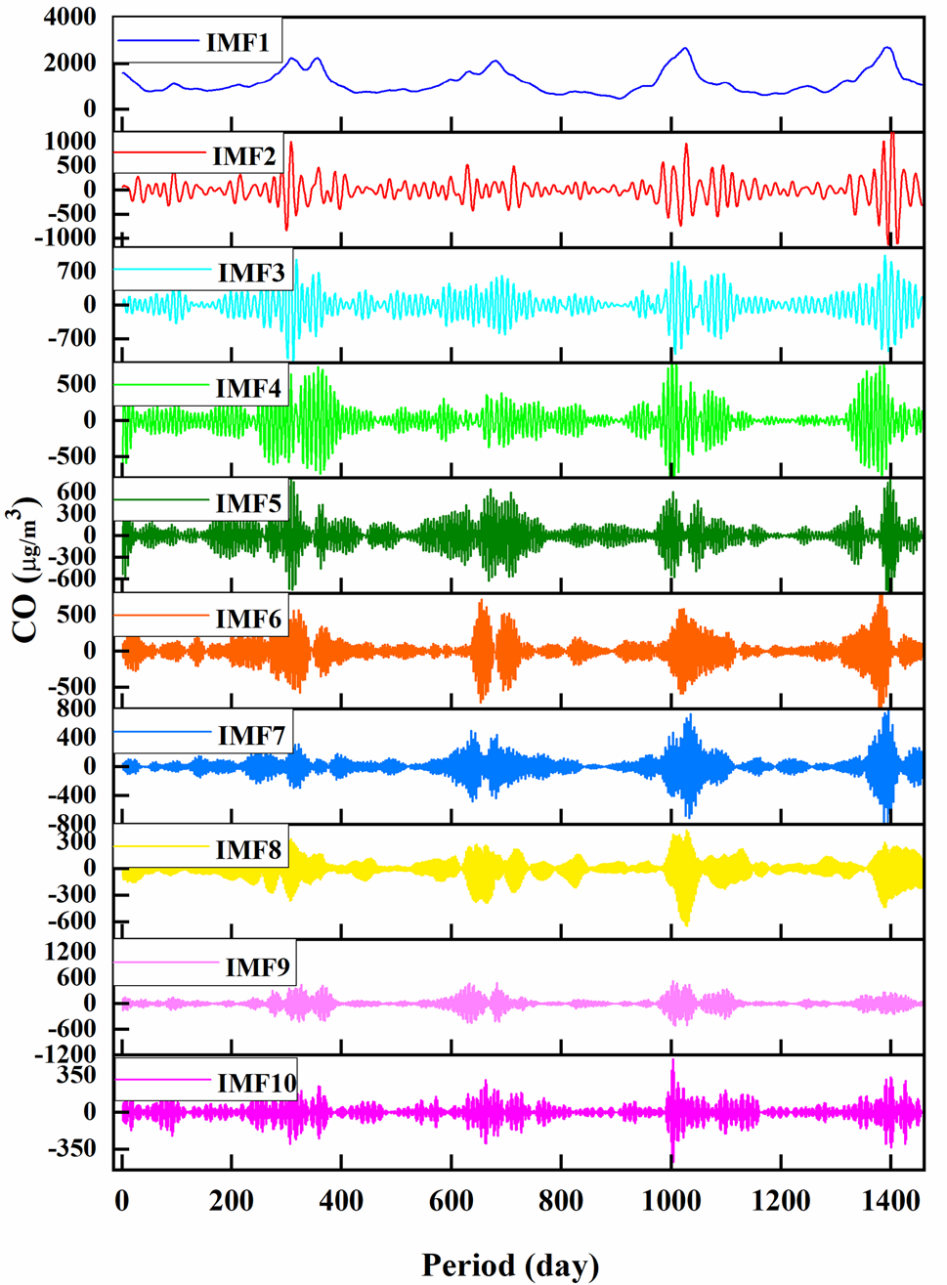
MVMD outputs (IMFs) for CO parameter.

At this phase, the MVMD method decomposed the time series of input parameters. The independent variables were decomposed into their constituent elements, and stored in IMFs. This study considered the optimum number of modes for input variables, assigned by the letter K. The value of K, which was arrived at by the process of trial and error, is 10. As an illustration of how MVMD preprocessing works, Fig. 3 depicts the disintegration components (also known as IMFs) for the CO. We considered the current time step, as well as three delays for each input. Therefore, it was calculated that there were 400 input variables or 10 × 4 × 10IMFs = 400.

### Phase 2: feature selection by catboost model

**Fig. 4:**
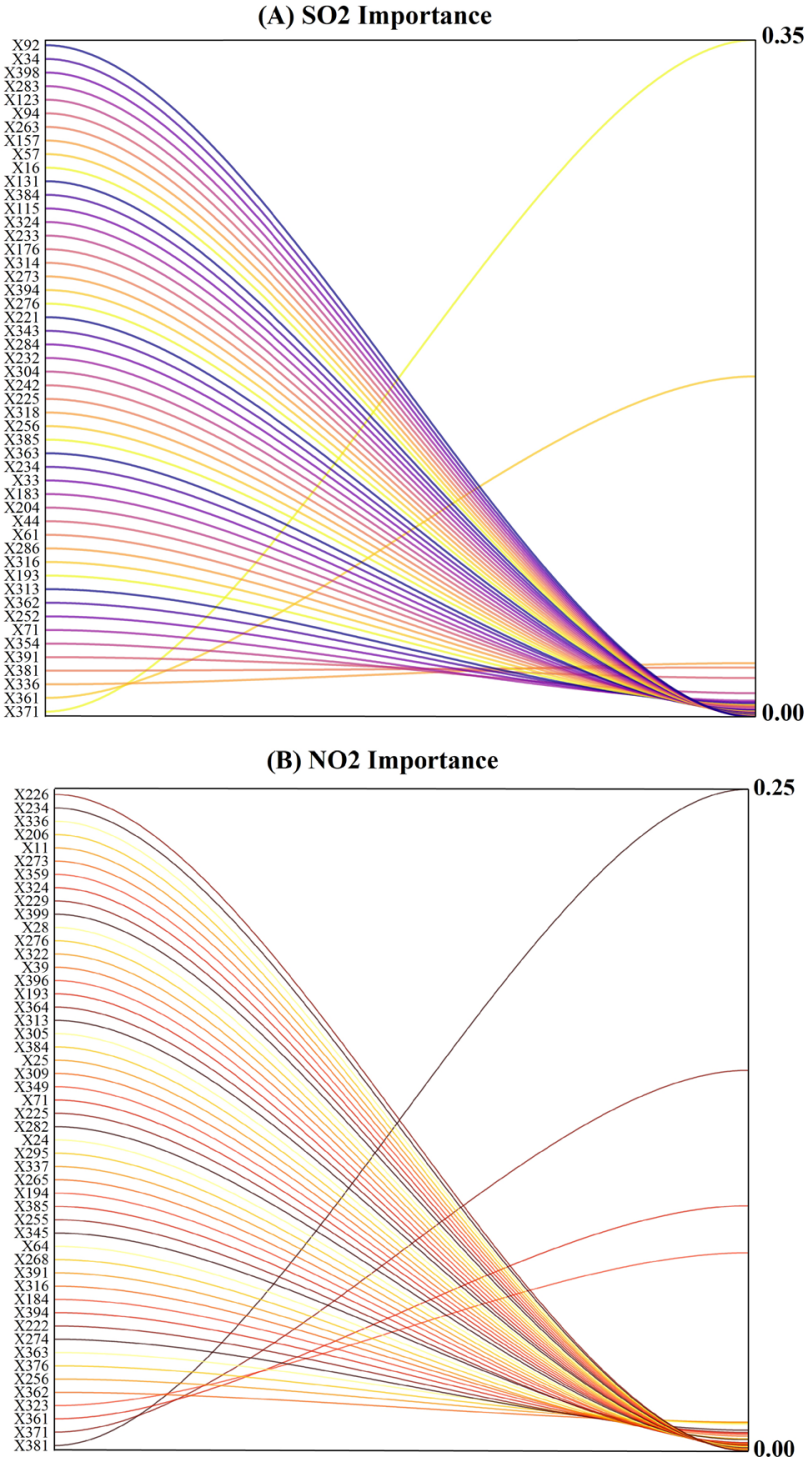
Feature selection using Catboost model to extract effective input variables to forecast **A** SO_2_ and **B** NO_2_.

Selecting influential features is one of the most critical processes involved in the development of ML models. As a consequence, applying feature selection approaches reduces the model’s computing cost and input dimension while increasing its precision and comprehension^27,28^. In this research, the MVMD method was used to decompose the input variables, and then the Catboost method as a feature selection, was employed to decrease the dimension of input variables. Figure 4 was a representation of the most significant decomposed characteristics that the Catboost model was able to produce. Based on the figure, about 35% of the input parameters were chosen (the number of input variables was set at 140) to forecast the SO_2_ (t + 1) and NO_2_ (t + 1) parameters. This section concludes that the Catboost technique eliminated around 65% of the input parameters that had been generated due to decomposing. As a result, this had a major impact on computation time and cost.

### Phase 3: accomplishment of ML models

The collected data was divided into two parts during the preprocessing procedures, specifically training and testing. This was performed to create ML-based models for SO_2_ and NO_2_ prediction. The ratio was divided into 75% for the training and 25% for the testing. In this study, five robust ML models were trained to predict SO_2_ and NO_2_. These methods included, LWKELM-ISA, LWLR-ISA, KELM-ISA, GPR-ISA and MARS-ISA. The dataset quantities were normalized to scale them between 0 and 1 during training, which had the quantity of input parameters. This is beneficial for faster convergence of the models. Steps two and three were omitted in the construction of standalone-based ML algorithms. Once the time series was normalized, all the input parameters could directly enter into ML methods. Accordingly, Eq. (32) is used to normalized the air quality dataset.

32 $$\:{X}_{N}=\frac{{X}_{F}-{X}_{MI}}{{X}_{MX}-{X}_{MI}}$$

where $\:{X}_{N}$ indicates normalized the air quality dataset, $\:{X}_{F}$ expresses measured value of air quality dataset, $\:{X}_{MI}$ and $\:{X}_{MX}$indicate the minimum and maximum values of dataset. To obtain the best results from ML-based prediction methods, it is necessary to adjust the relevant control parameters^29–32^. In this context, the ISA optimization approach was applied to obtain optimum tuning settings.

**Fig. 5:**
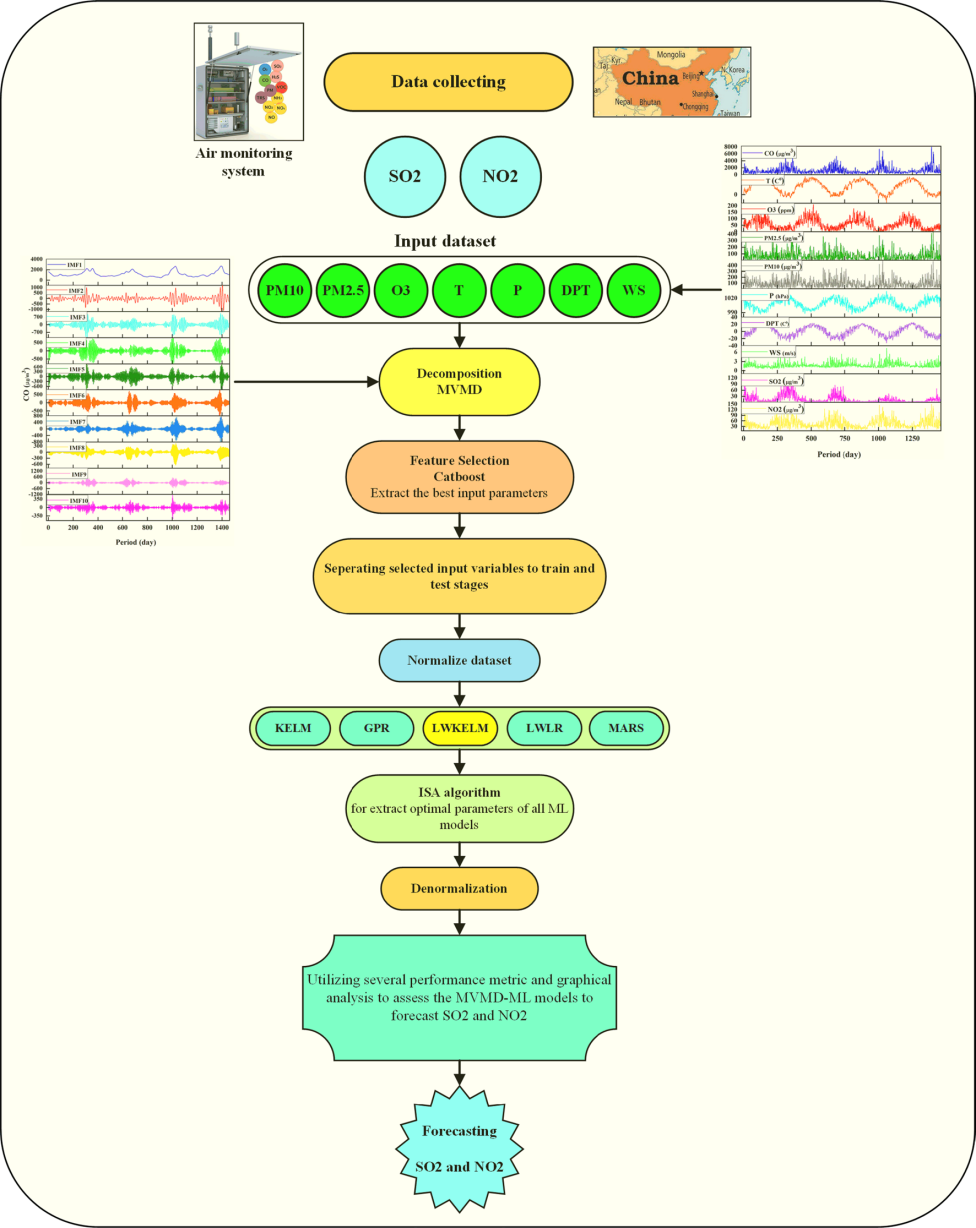
Schematic representation of the modeling framework for SO₂ and NO₂ prediction using multiple machine learning models.

To attain the most significant practical level of precision, the root-mean-squared error (RMSE) was employed as a fitness function. Tables 2 and 3 list the ML and MVMD-ML control parameters optimized by the ISA algorithm to produce API forecasts. Figure 5 shows a schematic depiction of the interplay between the several models and forecasts daily SO_2_ and NO_2_ values in the Changping region of China. In the GPR model, the kernel function is a radial basis function ($\:exp\left({a}^{2}\right)\times\:{exp}\left(-\frac{{\left|{x}_{i}-{x}_{j}\right|}^{2}}{2\times\:{\left({exp}\left(b\right)\right)}^{2}}\right)$), and there are two tuning parameters for it (*a* and *b*).

**Table 2:** Amounts of control parameters for ML methods to forecast SO_2_.

Model	Models	Tuning parameter models
ML	LWKELM	$\:\nu\:=1.93E+09,\:\rho\:=2.00E+12,\:\mu\:=1.98E+12,\:C=8.85E+11$
	KELM	$\:\nu\:=1.00E-10,\:\rho\:=7.16E+11,\:\mu\:=6.64E+11,\:C=7.62E+09$
	LWLR	$\:\nu\:=5.88E+10,\:\rho\:=7.71E+11,\:\mu\:=7.91E-02$
	MARS	Number of BFs = 30
	GPR	$\:a=10.85,\:b=3.064$
MVMD-ML	LWKELM	$\:\nu\:=2.23E+10,\:\rho\:=1.86E+12,\:\mu\:=1.85E+12,\:C=4.86E+09$
	KELM	$\:\nu\:=1.00E-10,\:\rho\:=1.67E+12,\:\mu\:=1.82E+12,\:C=1.89E+12$
	LWLR	$\:\nu\:=1.00E-10,\:\rho\:=1.80E+12,\:\mu\:=2.28E+10$
	MARS	Number of BFs = 35
	GPR	$\:a=17.74,\:b=13.32$

**Table 3:** Amounts of control parameters for ML methods to forecast NO_2_.

Model	Models	Tuning parameter models
ML	LWKELM	$\:\nu\:=2.77E+10,\:\rho\:=1.98E+12,\:\mu\:=2.00E+12,\:C=6.92E+11$
	KELM	$\:\nu\:=1.00E-10,\:\rho\:=1.80E+12,\:\mu\:=2.72E+11,\:C=2.26E+09$
	LWLR	$\:\nu\:=1.00E-10,\:\rho\:=5.20E+10,\:\mu\:=8.22E+09$
	MARS	Number of BFs = 42
	GPR	$\:a=6.25,\:b=4.30$
MVMD-ML	LWKELM	$\:\nu\:=7.50E+08,\:\rho\:=1.43E+12,\:\mu\:=7.48E+11,\:C=1.04E+12$
	KELM	$\:\nu\:=1.00E-10,\:\rho\:=1.86E+12,\:\mu\:=8.80E+9,\:C=1.80E+09$
	LWLR	$\:\nu\:=1.39E+12,\:\rho\:=1.45E+12,\:\mu\:=1.00E-10$
	MARS	Number of BFs = 45
	GPR	$\:a=4.50,\:b=3.20$

## Results and discussions

We used 10 different ML models to predict one-day-ahead API values for this study. The reported models were the five single (LWKELM-ISA, KELM-ISA, LWLR-ISA, MARS-ISA and GPRISO) and five hybrid models (MVMD-LWKELM-ISA, MVMD-KELM- ISA, MVMD-LWLRIS A, MVMD-MARSI SA AND MV MD - G PRTSOlSA). The proposed ML models were trained with 1032 data of daily API variables, and then tested using 437 data. Their models’ performance was evaluated using six statistical performance measures (R, RMSE, NSE, IA, MaxAE, and U_95%_), investigated through graphical visual analysis (bar plot; marginal boxplot; scatter plot for basin-wise data; box plot of relative error; Taylor diagram). The most appropriate complementary ML model for ahead-of-time prediction would be the one with the lowest RMSE, MAPE and U95% while having a highest R, IA and NSE in testing stage. The results that were collected will be detailed in the subsequent subsections as follows:

### Comparison LWKELM-ISA with other ML methods

Tables 4 and 5 report the results of training and testing performed on standalone ML models to anticipate the SO_2_ and NO_2_ one day ahead of time. In the case of SO_2_, the findings of the testing stage indicated that the LWKELM-ISA approach yielded the best results in terms of R (0.680), NSE (0.244), and I_A_ (0.806), while also producing the lowest values for RMSE (7.369), MaxAE (46.151), and U95% (20.080). For the NO_2_ forecasting, Table 4 presents the results obtained by using the suggested LWKELM-ISA model, which had the greatest R (0.724), NSE (0.521), and IA (0.814) and the smallest RMSE (17.794), MaxAE (67.037), and U95% (49.319).

**Table 4:** Six statistical metrics to assess the single-based ML method to forecast SO_2_ (µg/m^3^).

Model	Metric	*R*	RMSE	NSE	I_A_	MaxAE	U_95%_
LWKELM	Train	0.849	10.809	0.720	0.912	50.083	29.969
	Test	**0.680**	**7.369**	**0.244**	**0.806**	**46.151**	**20.080**
MARS	Train	0.866	10.232	0.749	0.924	44.641	28.367
	Test	0.609	8.084	0.090	0.765	54.764	22.128
LWLR	Train	0.821	11.786	0.667	0.893	64.434	32.510
	Test	0.656	8.325	0.036	0.790	42.587	23.083
GPR	Train	0.820	11.687	0.673	0.892	62.601	32.403
	Test	0.640	8.251	0.052	0.779	42.231	22.509
KELM	Train	0.837	11.194	0.700	0.904	62.164	31.003
	Test	0.657	8.147	0.076	0.791	44.804	22.577

**Fig. 6:**
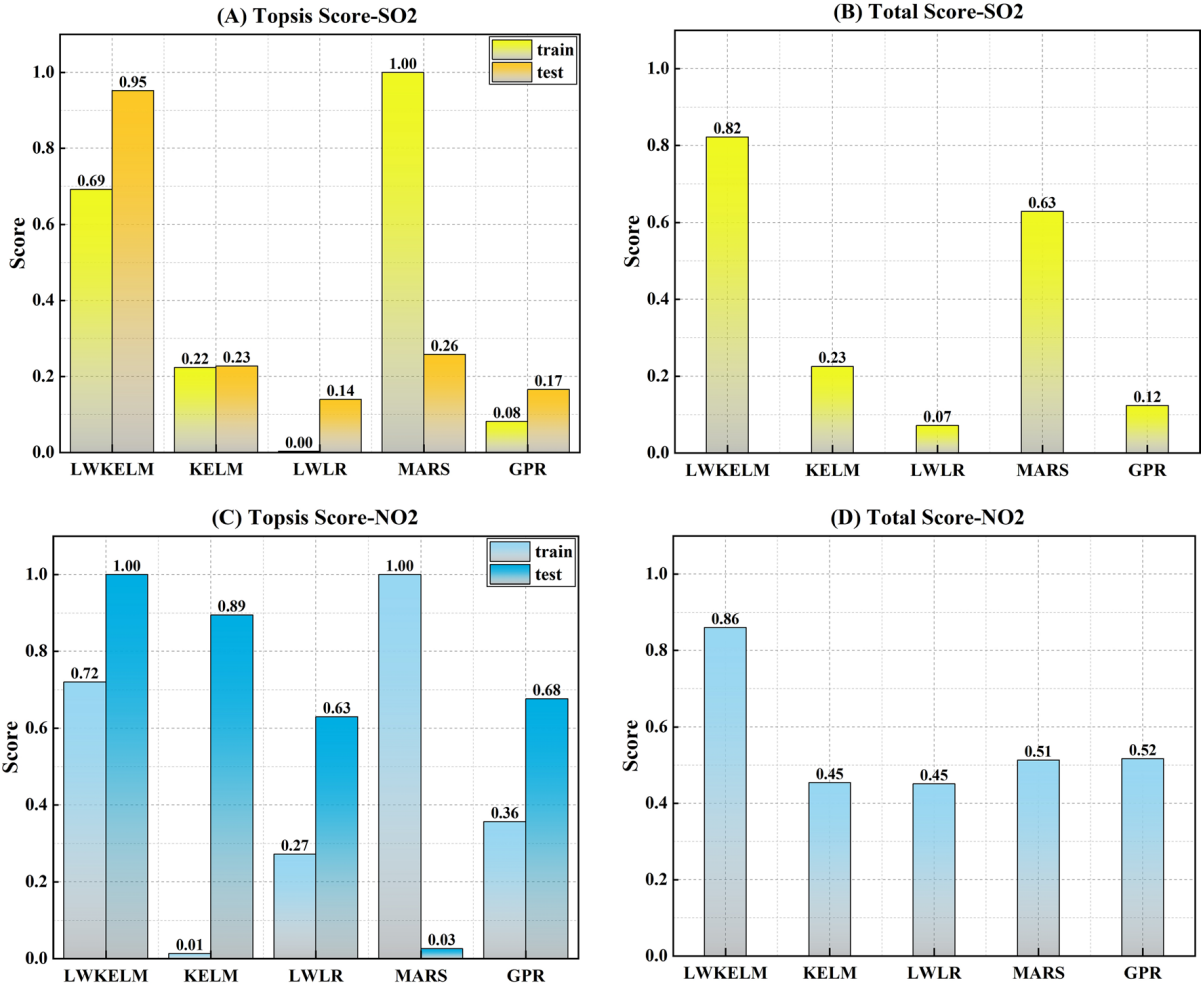
TOPSIS score achieved by single-based ML model to forecast SO_2_ and NO_2_.

Figure 6 shows a bar graph of the Topsis score for each ML method. From the figure, the LWKELM method, with a score equal to 0.69 and 0.95 in the training and testing stage, could provide the best efficiency. Additionally, the suggested approach had the highest efficiency at 0.82, followed by MARS at 0.63, KELM at 0.23, GPR at 0.12, and LWLR at 0.07. From Fig. 6C and D, the Topsis score of LWKELM-ISA (train: 0.72 and test: 1) was better than the other models. In addition, the total score of the proposed model (0.86) was higher than the GPR-ISA (0.52), MARS-ISA (0.51), LWLR-ISA (0.45), and KELM-ISA (0.45) models. Based on the results of this section, it is clear that no solo ML model could deliver enough accuracy. However, the MVMD approach could be used to improve the accuracy of all ML models in forecasting SO_2_ and NO_2_ parameters.

**Table 5:** Six statistical metrics to assess the single-based ML method to forecast NO_2_ (µg/m^3^).

Model	Metric	*R*	RMSE	NSE	I_A_	MaxAE	U_95%_
LWKELM	Train	0.744	14.842	0.554	0.839	68.125	41.150
	Test	**0.724**	**17.794**	**0.521**	**0.814**	**67.037**	**49.319**
MARS	Train	0.779	13.939	0.607	0.866	66.107	38.646
	Test	0.640	19.888	0.401	0.775	123.046	55.085
LWLR	Train	0.678	16.530	0.447	0.806	74.923	45.813
	Test	0.650	19.839	0.404	0.784	75.017	54.878
GPR	Train	0.714	15.571	0.509	0.812	80.496	43.170
	Test	0.669	19.196	0.442	0.759	77.685	53.227
KELM	Train	0.681	16.531	0.447	0.809	87.278	45.736
	Test	0.705	18.290	0.494	0.800	68.917	50.685

### Comparison MVMD-LWKELM-ISA with other MVMD-ML models

#### Statistical metrics analysis

In this part, the proposed MVMD-LWKELM-ISA model was evaluated for its ability to predict SO_2_ and NO_2_ parameters in comparison to MVMD-KELM-ISA, MVMD-LWLR-ISA, MVMD-MARS-ISA, and MVMD-GPR-ISA. As demonstrated in Tables 6 and 7, the MVMD-ML models were used to predict SO_2_ and NO_2_ one day in advance. Table 6 presents the outcomes of the models to forecast SO_2_, with the MVM-D-LWKELM-ISA appearing to be the most precise due to its small RMSE (train = 2.672 and test = 1.965), U95% (train = 7.408 and test = 5.417), and MaxAE (train = 13.276 and test = 8.634) qualities and large R (train = 0.991 and test = 0.974), IA (train = 0.996 and test = 0.986), and NSE (train = 0.983 and test = 0.946) values. Based on the results of NO_2_ forecasting (Table 7), the proposed model provided promising results in the training stage (*R* = 0.98, RMSE = 4.445, NSE = 0.960, IA = 0.990, MaxAE = 16.676, and U_95%_ = 12.324) and testing stage (*R* = 0.978, RMSE = 5.372, NSE = 0.956, IA = 0.989, MaxAE = 25.018, and U_95%_ = 14.882) compared with the other models.

**Table 6:** Six statistical metrics to assess the MVMD-based ML method to forecast SO_2_ (µg/m^3^).

Model	Metric	*R*	RMSE	NSE	I_A_	MaxAE	U_95%_
LWKELM	Train	0.991	2.672	0.983	0.996	13.276	7.408
	Test	**0.974**	**1.965**	**0.946**	**0.986**	**8.634**	**5.417**
MARS	Train	0.986	3.349	0.973	0.993	15.684	9.284
	Test	0.962	2.342	0.924	0.980	9.401	6.472
LWLR	Train	0.990	2.909	0.980	0.995	17.164	8.064
	Test	0.947	2.858	0.886	0.971	10.977	7.789
GPR	Train	0.979	4.248	0.957	0.989	24.234	11.778
	Test	0.929	3.243	0.854	0.963	18.289	8.943
KELM	Train	0.987	3.256	0.975	0.994	18.156	9.027
	Test	0.965	2.385	0.921	0.979	10.564	6.415

**Table 7:** Six statistical metrics to assess the MVMD-based ML method to forecast NO_2_ (µg/m^3^).

Model	Metric	*R*	RMSE	NSE	I_A_	MaxAE	U_95%_
LWKELM	Train	0.980	4.445	0.960	0.990	16.676	12.324
	Test	**0.978**	**5.372**	**0.956**	**0.989**	**25.018**	**14.882**
MARS	Train	0.977	4.742	0.954	0.988	16.977	13.149
	Test	0.966	6.744	0.931	0.983	25.615	18.702
LWLR	Train	0.978	4.655	0.956	0.989	18.860	12.902
	Test	0.965	6.787	0.930	0.982	31.943	18.788
GPR	Train	0.962	6.332	0.919	0.979	21.917	17.278
	Test	0.970	6.258	0.941	0.985	31.708	17.295
KELM	Train	0.963	6.107	0.925	0.980	20.049	16.824
	Test	0.968	6.510	0.936	0.983	31.646	18.037

**Fig. 7:**
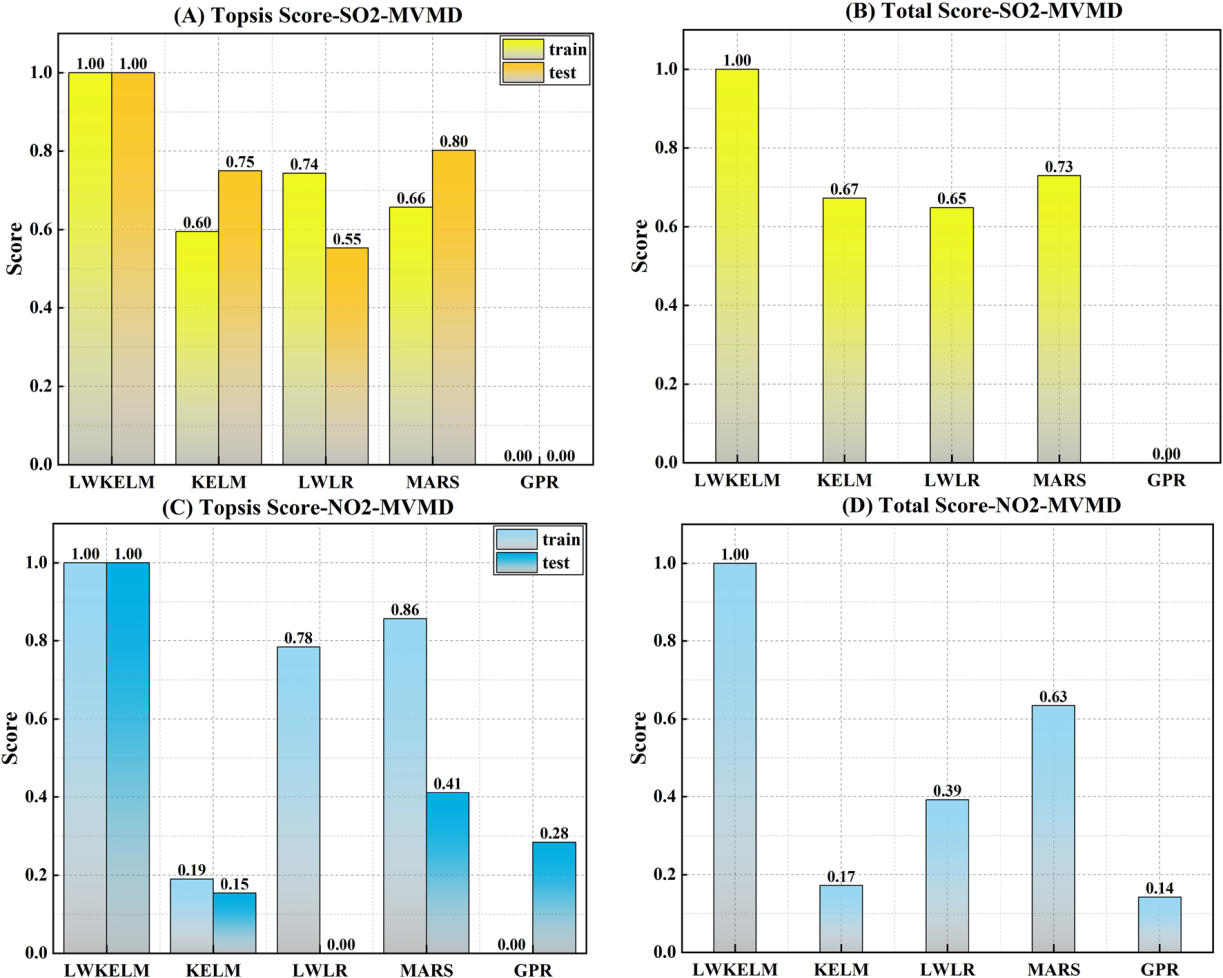
Topsis score achieved by MVMD-based ML model to forecast SO_2_ and NO_2_.

Figure 7 is a bar chart displaying the Topsis score for all ML models to forecast SO_2_ parameter. The LWKELM model receiving a score of 1 in both the training and testing phases seems to be the most effective. A total score of 1 indicates that the suggested model was the most effective, followed by MARS (0.73), KELM (0.67), LWLR (0.65), and GPR (0.00). In comparison to the other models, LWKELM-ISA had a higher Topsis score (train: 1, test: 1) to forecast the NO_2_ parameter, as shown in Fig. 7C and D. The suggested model also had a higher overall score (1) than the MARS-ISA (0.63), LWLR-ISA (0.51), KELM-ISA (0.17), and GPR-ISA (0.14).

#### Convergence analysis

**Fig. 8:**
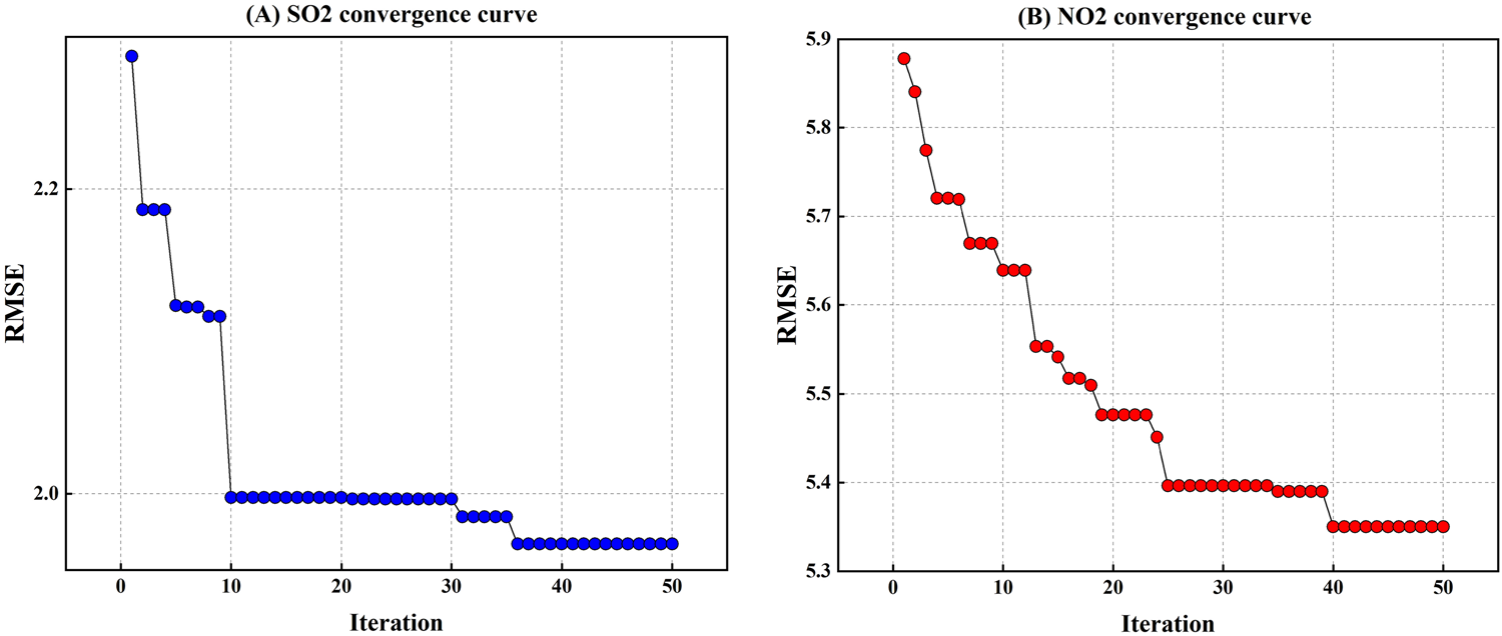
Convergence graph for **A** SO_2_ and **B** NO_2_ forecasts using ISA algorithm.

Figure 8a and b displayed the convergence curves of the ISA optimization method for the LWKELM model for SO_2_ and NO_2_, respectively. The figure indicates a stable and efficient optimization process. For SO_2_, the RMSE rapidly decreases in the initial iterations and gradually stabilizes around 1.96 after approximately 35 iterations, demonstrating that the algorithm quickly approaches the optimal solution. Similarly, for NO2, the RMSE declines steadily from 5.88 to around 5.37 over 50 iterations, showing a consistent improvement in prediction accuracy. Consequently, these convergence curves confirm that the ISA method effectively optimizes the LWKELM model, achieving low error values for both SO_2_ and NO_2_ forecasts.

#### Wilcoxon test analysis

Table 8 shows the Wilcoxon test results for SO₂ and NO₂ predictions for five ML methods. For SO₂, all models show statistically significant differences, with LWKELM (P-value = 1.21 × 10⁻¹²) and KELM (*p* = 3.70 × 10⁻⁷) showing the strongest significance, indicating that these models perform better. For NO₂, LWKELM (P-value = 7.97 × 10⁻⁴), KELM (*p* = 1.54 × 10⁻²), and GPR (*p* = 1.29 × 10⁻³) show statistically significant differences, indicating these models perform better in predicting NO₂. In contrast, LWLR (*p* = 0.76) and MARS (*p* = 0.83) show no significant differences, suggesting weaker performance. In conclusion, LWKELM appears to be the best performing model for SO₂, while for NO₂, GPR shows the most significant improvement.

**Table 8:** Wilcoxon test results for SO2 and NO2.

	SO_2_	
	Wilcoxon Statistic	*P*-value
Model	3.83E + 04	3.16E-04
LWKELM	2.91E + 04	1.21E-12
KELM	3.44E + 04	3.70E-07
LWLR	4.09E + 04	8.39E-03
MARS	4.07E + 04	6.42E-03
GPR	3.83E + 04	3.16E-04

	NO_2_	
Model	Wilcoxon Statistic	*P*-value
LWKELM	2.58E + 05	7.97E-04
KELM	2.15E + 05	1.54E-02
LWLR	2.57E + 05	7.60E-01
MARS	2.58E + 05	8.28E-01
GPR	1.88E + 05	1.29E-03

#### Scatter plot analysis

**Fig. 9:**
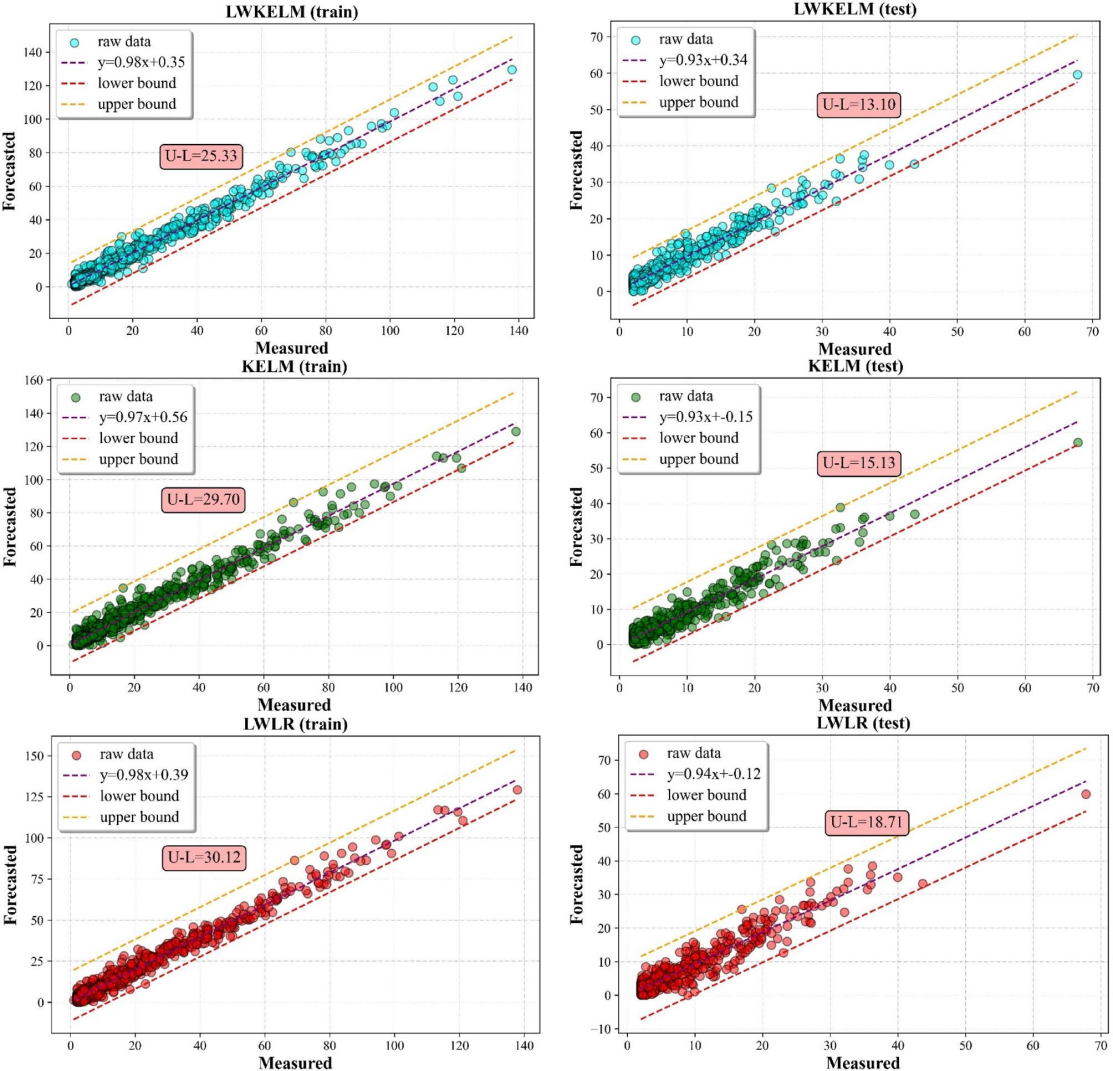

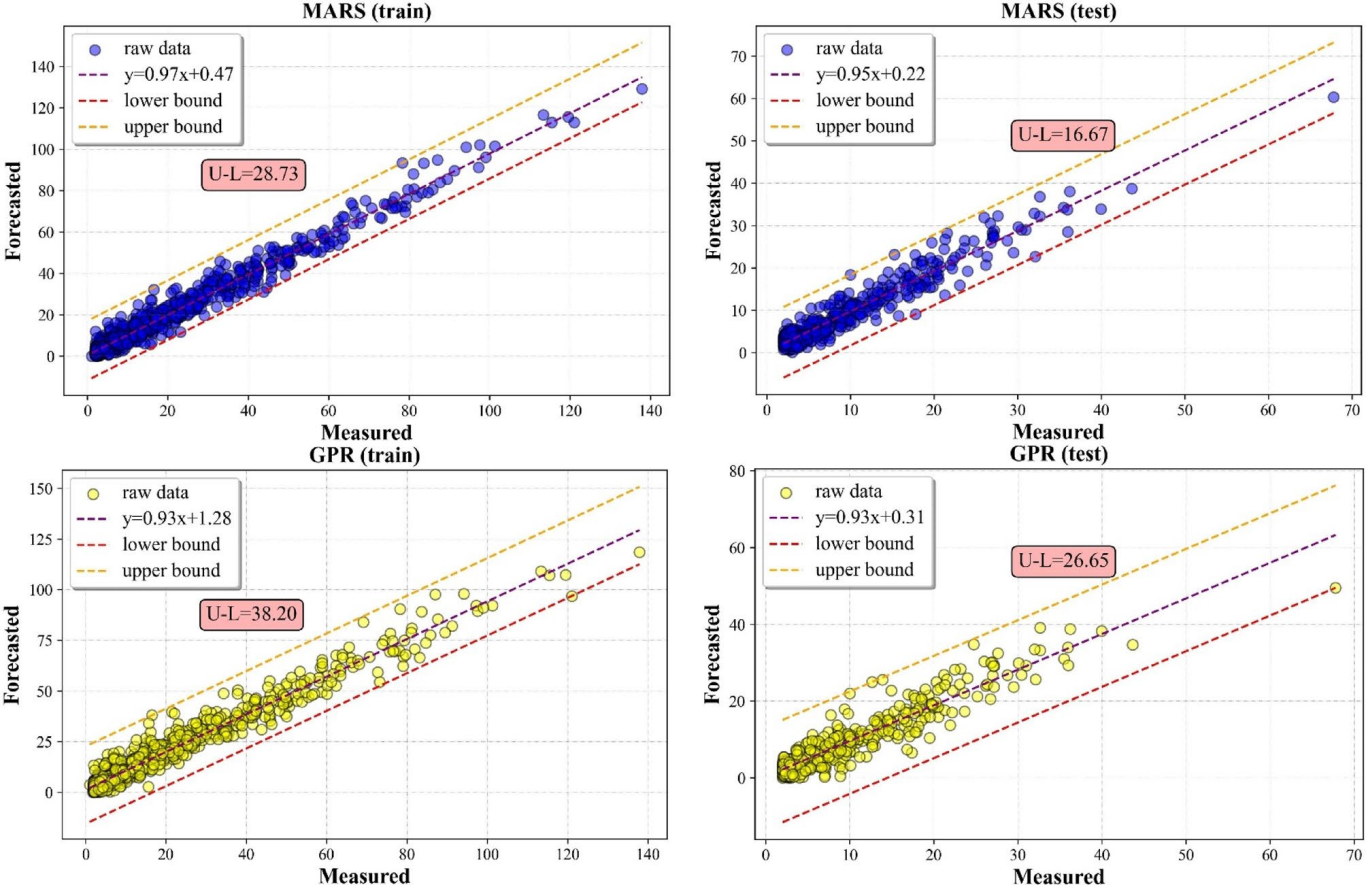
Scatter plots of all ML models to forecast SO_2_.

**Fig. 10:**
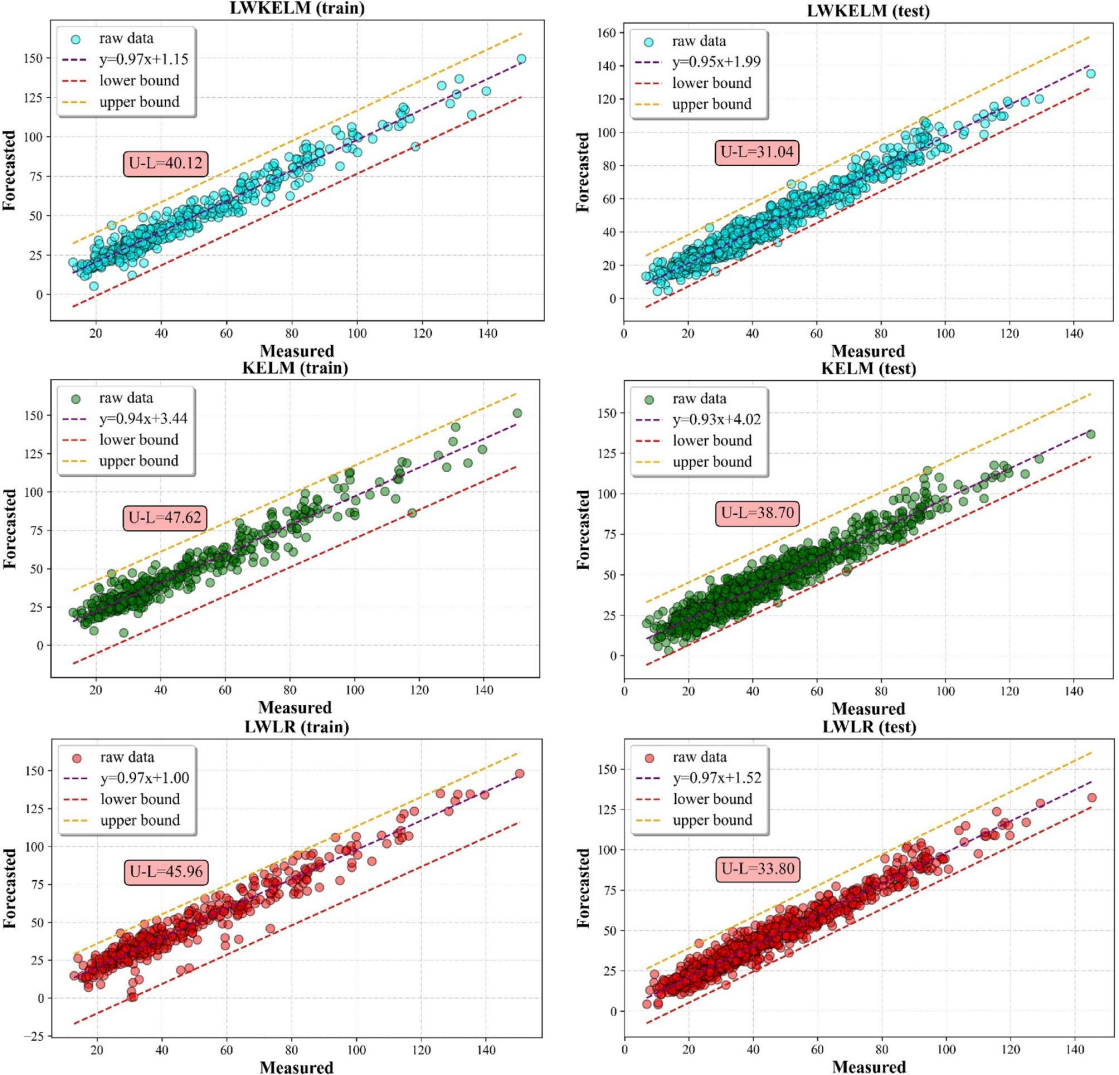

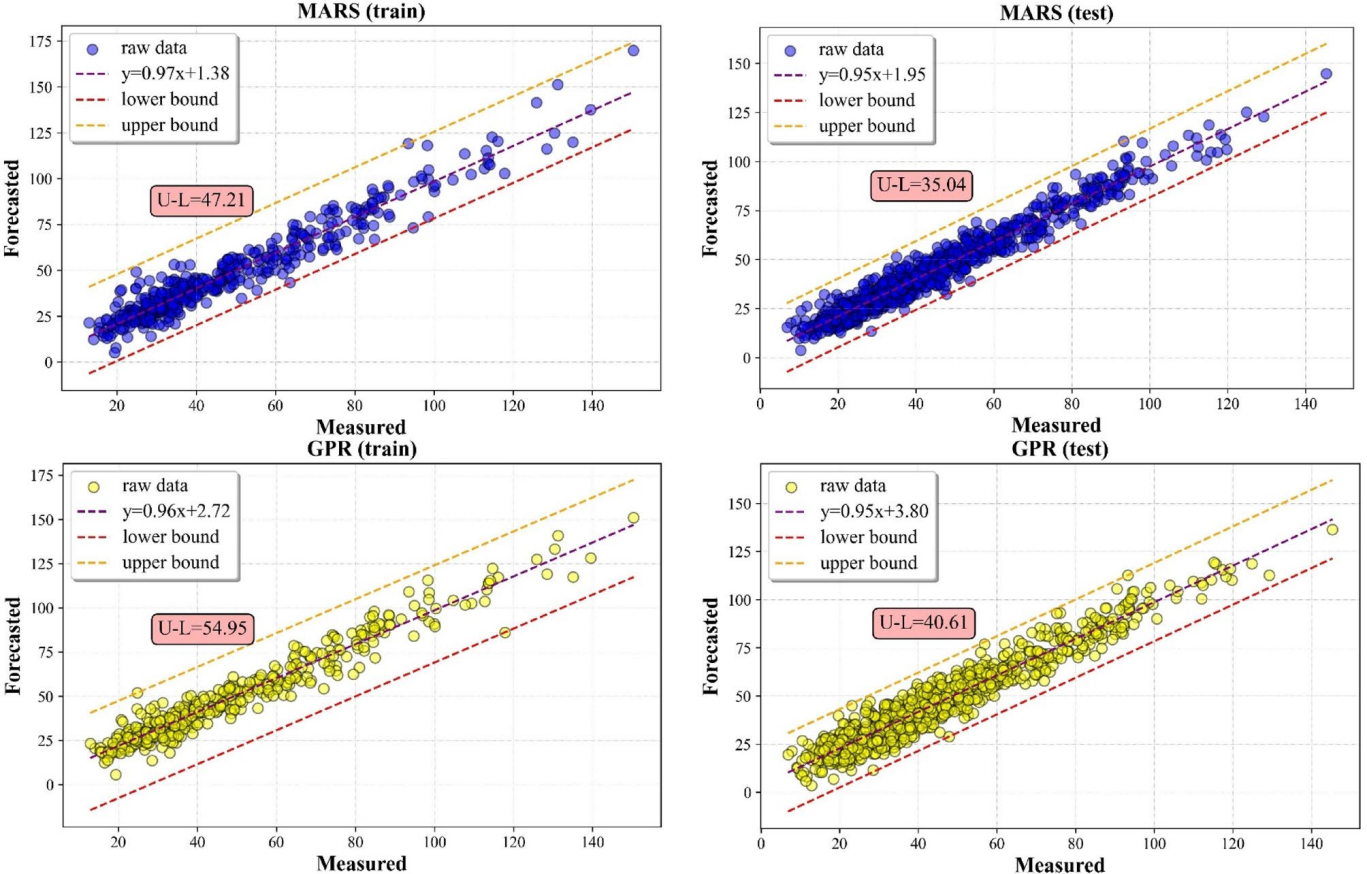
Scatter plots of all ML models to forecast NO_2_.

The results of the predictions for the SO_2_ and NO_2_ are shown in Figs. 9 and 10, using a scatter plot. Figures also showed the minimum and maximum values of a simulated distributed dataset for illustrating the discrepancy among other five MVMD-ML methods. In this aspect, a model is more consistent if its lower and upper bounds are tighter than those from other techniques. From the results, it can be seen that the MVMD-LWKELM-ISA model had better performance compared with those of benchmark methods, namely, the MVMD-KELM-ISA model, the MVMD-LWLR-ISA model, the MVMD-MARS-ISA model and the MVMD-GPR-ISA model. The results for SO_2_ forecast revealed that the difference between upper and lower bound (U–L) for MVMD-LWKELM-ISA (train: 25.33; test: 13.10) was less than that of MVMD-KELM-ISA (train: 29.70; test: 15.13), MVMD-MARS-ISA (train: 28.73; test: 16.67), MVMD-LWLR-ISA (train: 30.12; test:18.71) and MVMD-GPR-ISA (train:38.20; test :26.65). For NO_2_ prediction, the lowest U-L was obtained by our proposed model (train: 40.12 and test: 31.04), followed by MVMD-LWLR-ISA (train: 45.96 and test: 33.80), MVMD-MARS-ISA (train: 47.21 and test: 35.04), MVMD-KELM-ISA (train: 47.62 and test of :38.70), and MVMD-GPR-ISA respectively (train = 54.95 and test = 40.61). Accordingly, the proposed model was proved to be more robust and accurate than other models.

#### Relative error analysis

**Fig. 11:**
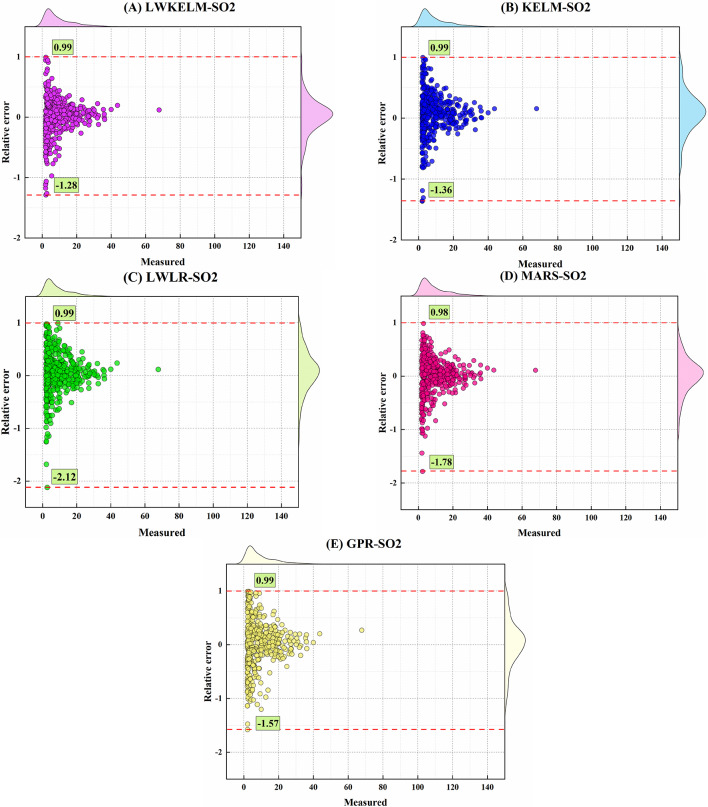
Marginal boxplot achieved by all model to forecast SO_2_.

**Fig. 12:**
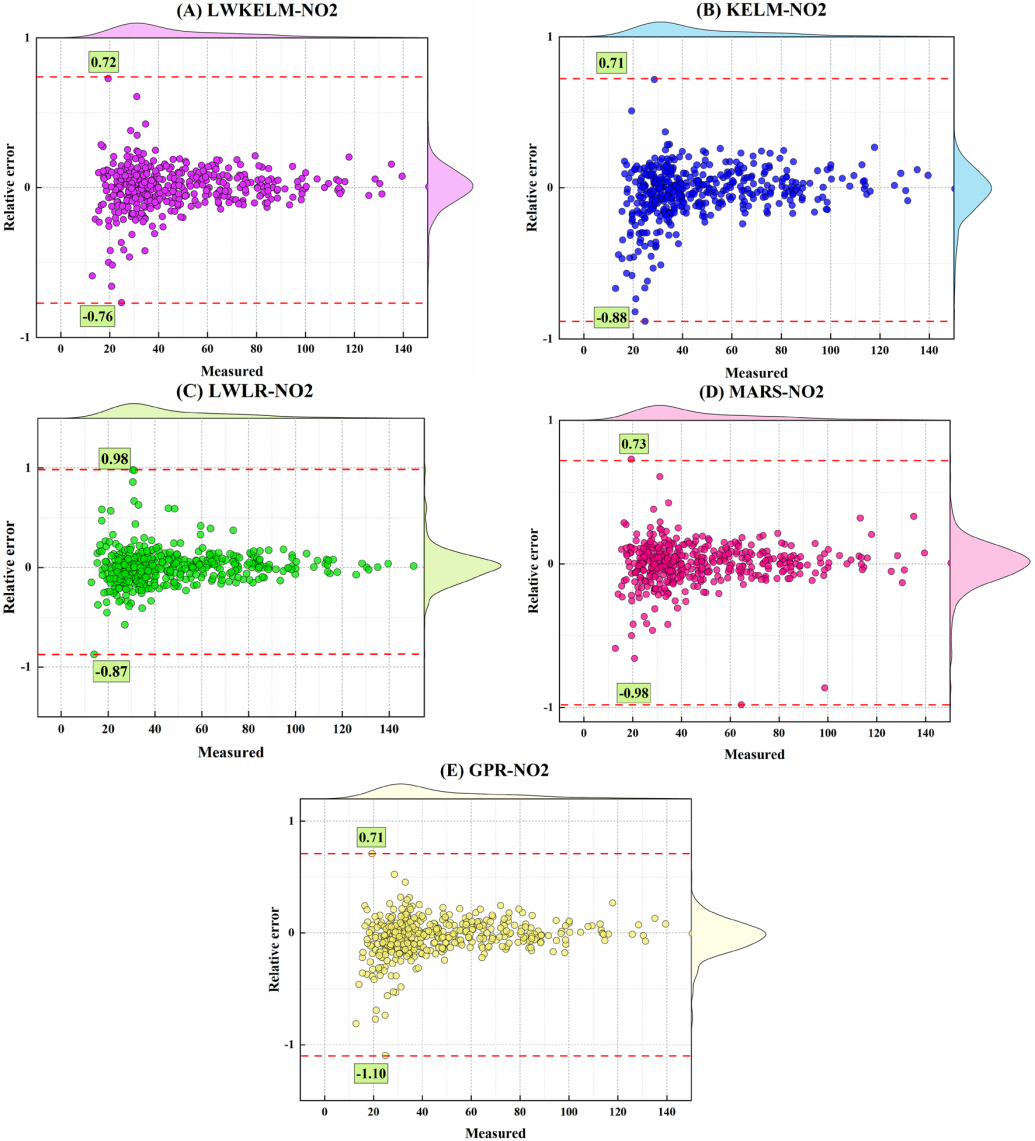
Marginal boxplot achieved by all model to forecast NO_2_.

Figures 11 and 12 depict the marginal boxplot of the relative error (RE) compared with the measured amounts of the SO_2_ and NO_2_ variables for the MVMD-ML models throughout the testing stages. For the SO_2_ parameter, the MVMD-LWKELM-ISA offered a much smaller range for RE ([−1.28, 0.99]) than the MVMD-KELM-ISA ([−1.36, 0.99]), MVMD-LWLR-ISA ([−2.12, 0.99]), MVMD-MARS-ISA ([−1.78, 0.98]), and MVMD-GPR-ISA ([−1.57, 0.99]). Figure 12 shows that the MVMD-LWKELM-ISA model outperformed the other MVMD-ML-based models to forecast NO_2_ in terms of accuracy throughout the testing phase, with the RE range equal to ([−0.76, 0.72]), ([−0.88, 0.71]), ([−0.87, 0.98]), ([−0.98, 0.73]), and ([−1.10, 0.71]) for MVMD-LWKELM-ISA, MVMD-KELM-ISA, MVMD-LWLR-ISA, MVMD-MARS-ISA, and MVMD-GPR-ISA, respectively.

**Fig. 13:**
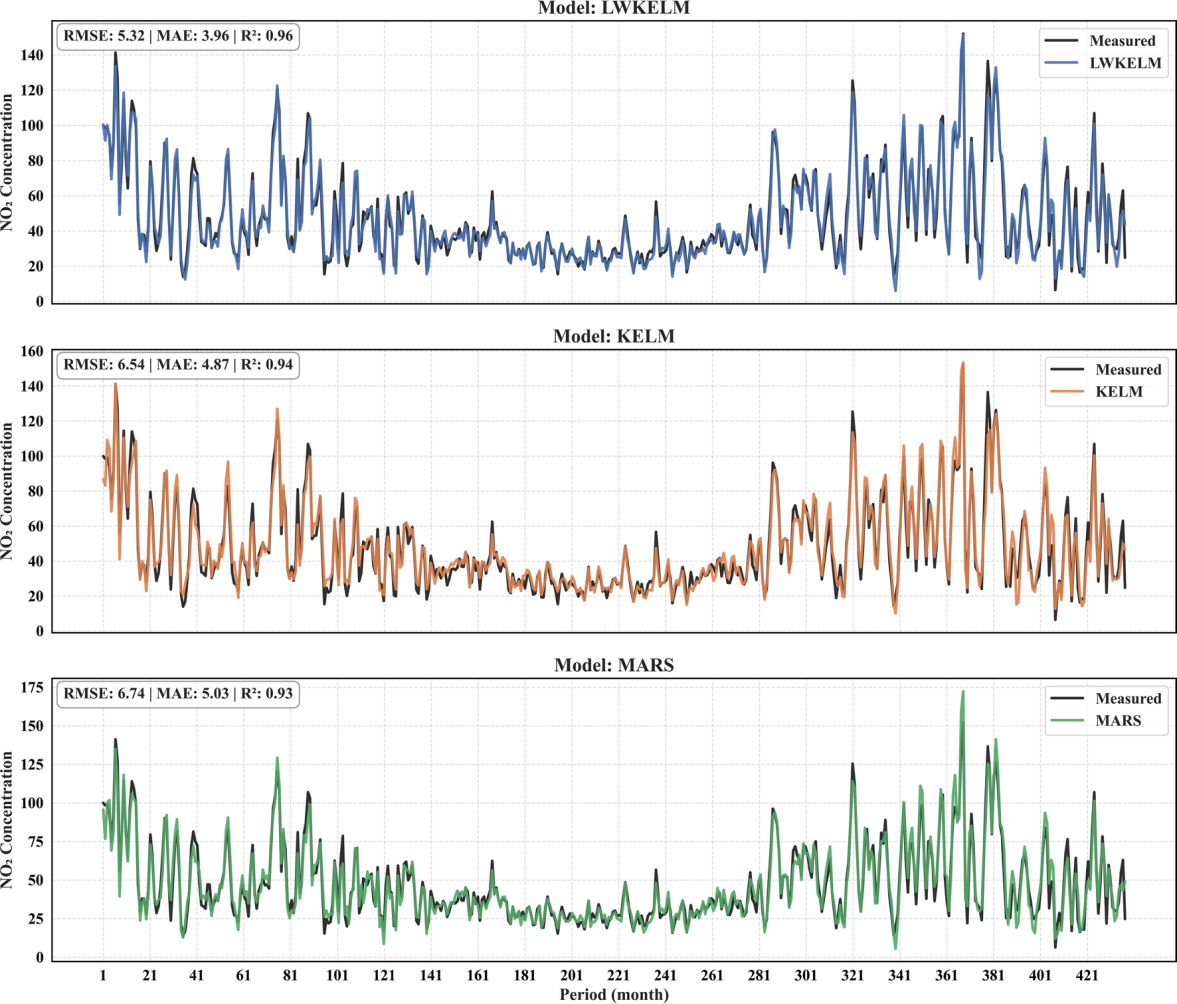
Time-series plot of measured versus forecasted values for **A** SO₂ and **B** NO₂ obtained by all ML models.

Figure 13A and B display the time-series plots of forecasted versus measured values of SO₂ and NO₂, respectively, during the testing stage. Figure 13A illustrates that all models reflected the general trend of SO₂ levels; yet, the LWKELM model demonstrated much greater concordance with the observed values. The LWKELM model achieved the lowest error values (RMSE = 1.94, MAE = 1.42) and the highest correlation coefficient (R² = 0.95), indicating its superior predictive capability. Conversely, the KELM (R² = 0.92) and MARS (R² = 0.93) models exhibited somewhat greater discrepancies from the empirical data. The findings affirm that the LWKELM model has the highest accuracy and reliability in predicting SO₂ concentration compared to the other models evaluated. Figure 13B shows the comparison between measured and predicted NO₂ concentrations from the LWKELM, KELM, and MARS models. All models captured the general trend, but LWKELM provided the best agreement with the measured data, showing the lowest errors (RMSE = 5.32, MAE = 3.96) and highest correlation (R² = 0.96). In contrast, KELM and MARS showed slightly larger deviations. Overall, LWKELM achieved the highest accuracy in forecasting NO₂ concentrations.

#### Taylor diagram analysis

**Fig. 14:**
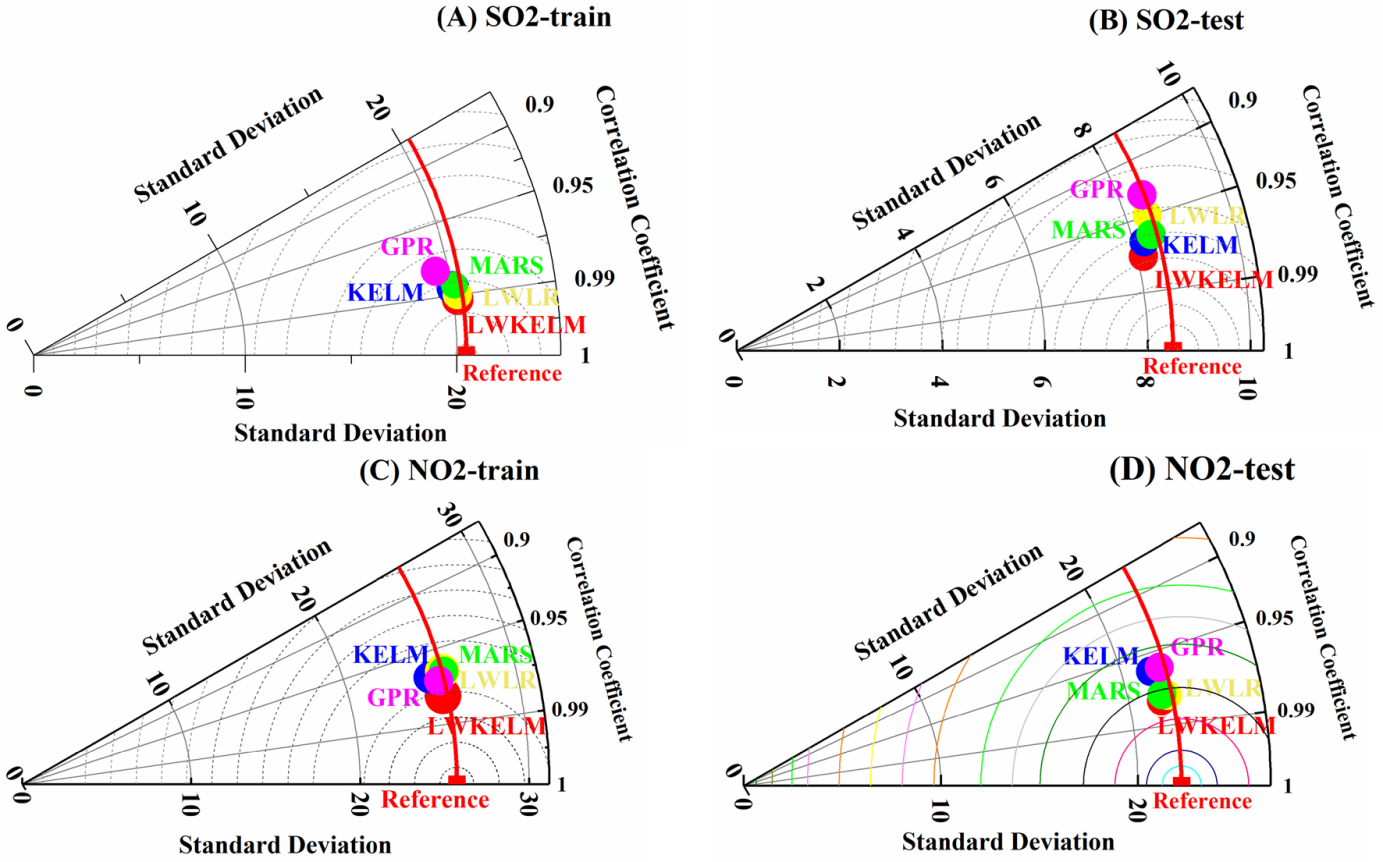
Taylor diagrams obtained by all models to forecast SO_2_ (**A** train and **B** test) and NO_2_ (**C** train and **D** test).

Figure 14 shows the Taylor diagrams of observed and predicted SO_2_ and NO_2_ concentrations for one day ahead in Changping. These diagrams were created over training and test sets for the MVMD-LWKELM-ISA, MVMD-KELM-ISA, MVMD-LWLR-ISA, MVMD-MARS-ISA and MVMD-GPR-ISA models. In general, the Taylor diagram is a plot of three statistical parameters: standard deviation (SD), correlation coefficient (R), and root mean squared error (RMSE)^33^. An alternative method of measuring the model’s precision is to compare how similar the test field is to the reference field. In fact, the models with the highest similarity to the reference point will be considered the most reliable. The reference point was represented by the red square on the x-axis of the figure. According to the figure, SO_2_ and NO_2_ were forecast better by LWKELM model than other models as they were nearer to reference point. From the results, it is evident that the LWKELM model can significantly improve the prediction performance of SO_2_ and NO_2_ compared with other methods.

#### SHAP explanation

**Fig. 15:**
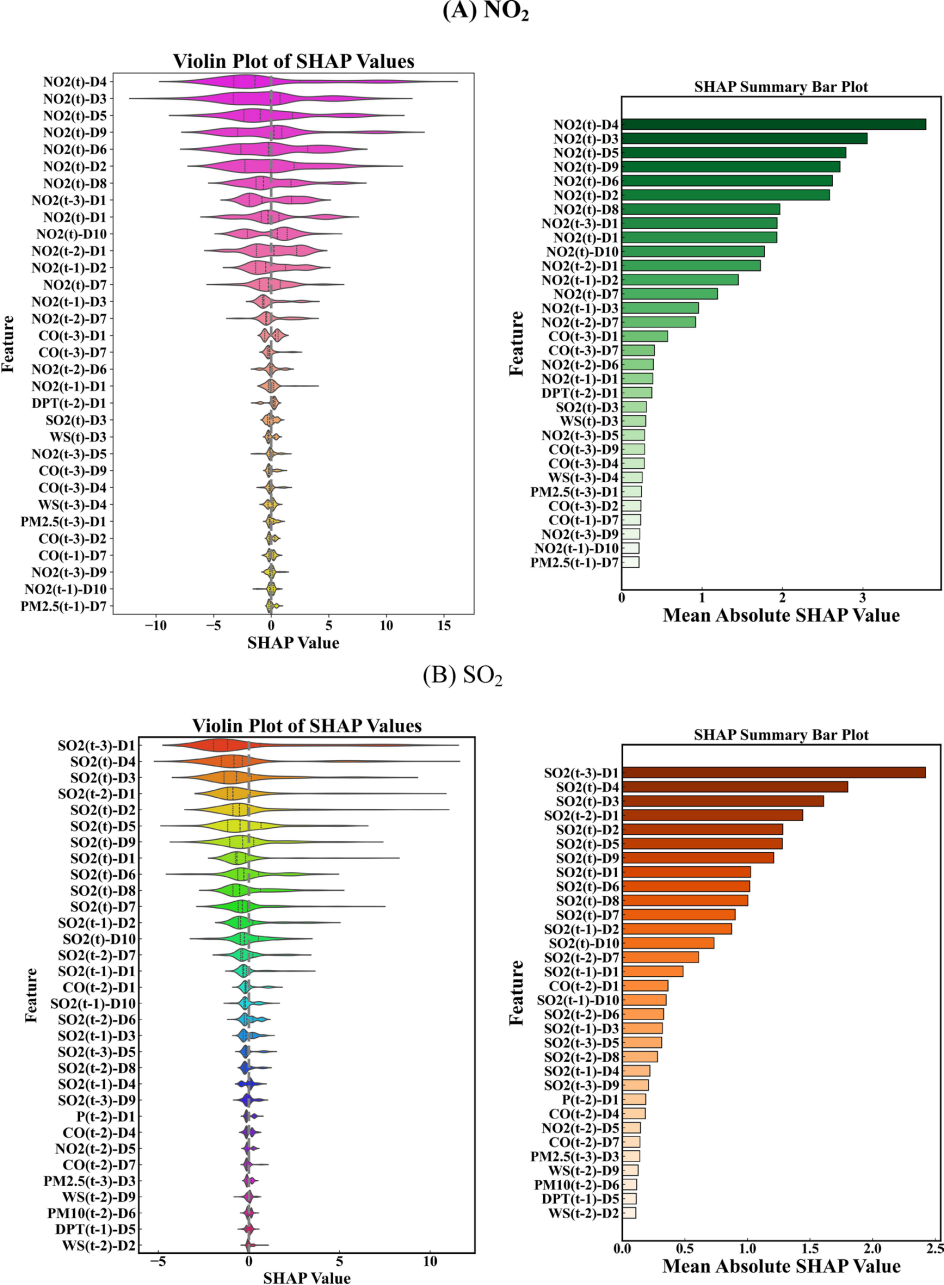
SHAP explanation analysis for simulated **A** NO_2_ and **B** SO_2_.

Figure 15A shows a comprehensive SHAP analysis to examine the effects of different features on the model predictions for NO₂. In the left panel of plot (A), the violin plot visualizes the distribution of SHAP values ​​for each feature and shows how much they influence the model predictions. The width of each violin reflects the density of SHAP values ​​and shows how specific features such as NO₂(t)-D4 and NO₂(t)-D3 have a wide range of effects on the predictions, indicating the variability in their impact. Meanwhile, right panel of plot (A) presents a bar chart that ranks features based on their mean absolute SHAP values. Here, NO₂(t)-D4 is ranked as the most important feature, meaning that its changes have a significant impact on the model outputs. This emphasizes the key role of decomposition level 4 (D4) in determining NO₂ levels and highlights the importance of selecting effective features for accurate predictive modeling.

Figure 15B presents a detailed SHAP analysis to indicate the impact of various features on the model predictions for SO₂. In the left panel of plot (A), the width of each violin indicates the density of SHAP values and demonstrates that features such as SO₂(t-3)-D1 and SO₂(t-4) show a considerable range of effects on predictions. The right panel of plot (A) presents a bar chart that ranks the features based on their mean absolute SHAP values. The figure shows that SO₂(t-3)-D1 is the most important feature, indicating that changes in this feature significantly impact the model’s results. This analysis highlights the crucial role of the time-lagged decomposition level (D1) in forecasting the SO₂ parameter.

## Conclusion

Due to the fast growth of industry, an accurate forecast of urban air pollution has emerged as a crucial early warning aspect to protect people’s health. A cutting-edge model of machine learning has been designed to achieve superior accuracy in predicting. In this research, the LWKELM model was developed as a combination of the KELM model with a set of weights. Applying these weights to the input data improved their ability to forecast the SO_2_ and NO_2_ parameters. The ISA algorithm, a robust optimization approach, was used to derive optimum parameters for the LWKELM model. The ISA made use of a reliable global search and local search technique to carry out an adequate global search during the first iteration and a precise search throughout the last iterations.

In this research, multiple input parameters (i.e., PM_10_, PM_2.5_, CO, O_3_, WS, P, T, and DPT) were utilized to forecast SO_2_ and NO_2_. In the first stage, we employed the MVMD technique to break down the input parameters into IMFs. The next stage was to select high-quality features by employing the Catboost method. The selected characteristics were then included in the LWKELM model to generate a forecast for one day ahead of SO_2_ and NO_2_ for a station in Changping. The effectiveness of the suggested approach was evaluated using a total of four different machine learning models, namely KELM, LWLR, MARS, and GPR.

Based on the results of the research, it was evident that the MVMD-LWKELM-ISA model was superior to the MVMD-KELM-ISA, MVMD-LWLR-ISA, MVMD-MARS-ISA, and MVMD-GPR-ISA models for forecasting SO_2_ and NO_2_ parameters. The suggested model’s findings for SO_2_ forecasting (*R* = 0.974, RMSE = 1.965, MaxAE = 8.634, U_95%_ = 5.417) were more precise than the other models. Also, to forecast NO_2_, the results confirmed the high precision of the proposed model in terms of several statistical metrics (i.e., *R* = 0.978, RMSE = 5.372, MaxAE = 16.676, U_95%_ = 14.882). In addition, the graphical analysis (i.e., scatter plot, marginal box plot of relative error, box plot, and Taylor diagram) demonstrated that the proposed MVMD-LWKELM-ISA model was superior to the other models to forecast SO_2_ and NO_2_ parameters. The innovative framework created in this study is unique because it combines the MVMD, Catboost, and ISA techniques with the LWKELM model, significantly enhancing forecasting accuracy.

To expand the scope of the MVMD-LWKELM-ISA, the proposed framework could address other emerging areas, such as environmental challenges, floods, drought, agriculture, and energy demand. This leads us to ascertain that the proposed model is highly suitable for practical applications, such as the pollution monitoring system that delivers dependable forecasts of air quality to safeguard public health. Moreover, the incorporation of more advanced feature-selection methodologies or hybrid dimensionality-reduction strategies may contribute to the reduction of redundancy and the augmentation of generalization capabilities. The application of advanced decomposition techniques or adaptive multiscale fusion methodologies may bolster the model’s proficiency in discerning high-frequency variations, particularly concerning pollutants such as NO₂. In conclusion, the proposed model exhibits significant potential for practical application, particularly within pollution monitoring frameworks that provide accurate air quality assessments to safeguard public health. In future work, it is reecommended to incorporate the multi-step forecasting to extend prediction horizons, explore additional decomposition methods for better feature interpretability, and implement uncertainty quantification techniques like Bayesian inference to enhance prediction reliability.

## Data Availability

No datasets were generated or analysed during the current study.
